# FOXO1 and GSK-3β Are Main Targets of Insulin-Mediated Myogenesis in C2C12 Muscle Cells

**DOI:** 10.1371/journal.pone.0146726

**Published:** 2016-01-19

**Authors:** Anna Litwiniuk, Barbara Pijet, Maja Pijet-Kucicka, Małgorzata Gajewska, Beata Pająk, Arkadiusz Orzechowski

**Affiliations:** 1 Department of Neuroendocrinology, Centre of Postgraduate Medical Education, Marymoncka 99/103, 01–813, Warsaw, Poland; 2 Department of Molecular and Cellular Neurobiology, Laboratory of Neurobiology, Nencki Institute of Experimental Biology PAS, Pasteura 3, 02–093, Warsaw, Poland; 3 Department of Dermatology, Medical University of Warsaw, Koszykowa 82A, 02–008, Warsaw, Poland; 4 Department of Physiological Sciences, Warsaw University of Life Sciences–SGGW, Nowoursynowska 159, 02–776, Warsaw, Poland; 5 Electron Microscopy Platform, Mossakowski Medical Research Centre PAS, Pawińskiego 5, 02–106, Warsaw, Poland; University of Minnesota Medical School, UNITED STATES

## Abstract

Myogenesis and muscle hypertrophy account for muscle growth and adaptation to work overload, respectively. In adults, insulin and insulin-like growth factor 1 stimulate muscle growth, although their links with cellular energy homeostasis are not fully explained. Insulin plays critical role in the control of mitochondrial activity in skeletal muscle cells, and mitochondria are essential for insulin action. The aim of this study was to elucidate molecular mechanism(s) involved in mitochondrial control of insulin-dependent myogenesis. The effects of several metabolic inhibitors (LY294002, PD98059, SB216763, LiCl, rotenone, oligomycin) on the differentiation of C2C12 myoblasts in culture were examined in the short-term (hours) and long-term (days) experiments. Muscle cell viability and mitogenicity were monitored and confronted with the activities of selected genes and proteins expression. These indices focus on the roles of insulin, glycogen synthase kinase 3 beta (GSK-3β) and forkhead box protein O1 (FOXO1) on myogenesis using a combination of treatments and inhibitors. Long-term insulin (10 nM) treatment in “normoglycemic” conditions led to increased myogenin expression and accelerated myogenesis in C2C12 cells. Insulin-dependent myogenesis was accompanied by the rise of *mtTFA*, *MtSSB*, *Mfn2*, and mitochondrially encoded *Cox-1* gene expressions and elevated levels of proteins which control functions of mitochondria (kinase—PKB/AKT, mitofusin 2 protein—Mfn-2). Insulin, via the phosphatidylinositol 3-kinase (PI3-K)/AKT-dependent pathway reduced transcription factor FOXO1 activity and altered GSK-3β phosphorylation status. Once FOXO1 and GSK-3β activities were inhibited the rise in *Cox-1* gene action and nuclear encoded cytochrome *c* oxidase subunit IV (COX IV) expressions were observed, even though some mRNA and protein results varied. In contrast to SB216763, LiCl markedly elevated Mfn2 and COX IV protein expression levels when given together with insulin. Thus, inhibition of GSK-3β activity by insulin alone or together with LiCl raised the expression of genes and some proteins central to the metabolic activity of mitochondria resulting in higher ATP synthesis and accelerated myogenesis. The results of this study indicate that there are at least two main targets in insulin-mediated myogenesis: notably FOXO1 and GSK-3β both playing apparent negative role in muscle fiber formation.

## Introduction

Skeletal muscle is the largest organ targeted by insulin in adult healthy individuals. Muscles often encompass more than 40% of the body weight, except in conditions of overweight, obesity or muscle cachexia. Consequently, muscles determine endurance to exercise as well as utilization of glucose. Adaptation to workload in skeletal muscle is however limited to available energy stores, which are highly dependent on aerobic metabolism in mitochondria. Impaired insulin activation of muscle glycogen synthase (GSK-3β) represents a consistent, molecular defect of the insulin signaling pathway [1–3]. We previously reported that insulin stimulates the metabolic differentiation of postnatal bovine skeletal muscle into muscle fibers type I slow-twitch oxidative [4]. In pathology, progressive loss of muscle mass is observed in diabetes, obesity and sarcopenia known as insulin resistant states [5–6]. Insulin-resistant states which weaken skeletal muscles are observed in several diseases accompanied by mitochondrial malfunction [7–8]. Previously, we reported that in “normoglycemic” conditions insulin stimulated mitochondriogenesis, and mitochondria were also required for insulin-mediated C2C12 muscle fiber formation [9]. For decade the efforts to reveal the molecular mechanisms underlying insulin resistance in human skeletal muscle were focused on possible links between glycogen synthase activity and mitochondrial dysfunction [10].

Insulin effects are provoked by several signaling cascades which are initiated at the level of the insulin receptor. Nevertheless, this critical step might be severely impaired in insulin-resistant states including hyperglycemia [11]. Consequently, numerous insulin-mediated metabolic effects associated with accelerated cell proliferation, increased viability and elevated protein synthesis/suppressed muscle proteolysis are blunted. Moreover, disrupted insulin signaling leads to reduced expression of mitochondrial genes [12]. In contrast, myogenesis is accompanied by extensive biogenesis of mitochondria and bioenergetic remodelling [13]. Furthermore, mitochondrial transcription factor A (Tfam), mitochondrial single-stranded DNA-binding protein (MtSSB), and nuclear respiratory factor 1 **(**NRF-1) are fundamental transcription factors in control of mitochondrial function. It is widely known, that biogenesis of mitochondria is orchestrated by peroxisome proliferator activated receptor-β co-activator-1α (PGC-1α), [14–17]. PGC-1α is also indirectly involved in regulating the expression of mtDNA transcription via increased expression of which is coactivated by NRF-1 [18–19]. Additionally, PGC-1α is downstream of FOXO, the forkhead type transcription factor (FKHR)[20]. FOXO1 was shown to be a negative regulator of skeletal muscle mass and expression of type I fiber-related genes [21–22]. FOXO1 transgenic mice showed poor glycemic control and low capacity for physical exercise [23]. Unlike, *Foxo1* haploinsufficiency restored insulin sensitivity and rescued the diabetic phenotype in insulin-resistant mice [24]. Paradoxically, FOXO1 also acts as an insulin sensor to activate insulin signaling, allowing a fast response to the hormone and vice versa [25]. Indeed, FOXO1 is a class of transcription factors that induce the atrophy-related ubiquitin ligase atrogin-1, but once phosphorylated by AKT upon insulin stimulation, FOXO1 cytoplasmic retention prevents atrogin-1-dependent proteolysis [26]. GSK-3β inhibition was frequently reported to be vital for muscle growth and differentiation [27–29] but its position in mitochondriogenesis during muscle differentiation is less clear.

Mitochondria are at the heart of metabolism and cell death, therefore they are important for many physiological pathways including both insulin secretion and signal transduction [30–31]. PGC-1α as a master integrator interacts with PPAR-γ to control activities of transcription factors that regulate mitochondriagenesis (i.e. NRF-1/2). As PGC-1α is repressed by FOXO1 [32], it is likely that insulin-dependent biogenesis of mitochondria might occur through NRF-1-mediated transcription of *mtTfa* as its downstream target [33–34]. Apparently, Tfam is the master regulator of mitochondrial biogenesis as it controls transcription and replication of mtDNA [35]. Increased mitochondrial-encoded cytochrome *c* oxidase (COX) subunits were observed during myogenesis at protein and mRNA levels, respectively [9, 36].

The aim of this study was to shed more light on the potential role of GSK-3β and FOXO1 in insulin-dependent control of mitochondriogenesis and myogenesis in normoglycemic *in vitro* conditions. To understand, what might be a possible molecular mechanism of insulin-mediated muscle fiber formation further study was undertaken in C2C12 myotubes with emphasis on the role played by Mfn-2 protein in muscle cell hypertrophy.

## Materials and Methods

### Materials

Media [Dulbecco’s modified Eagles medium (DMEM) low Glucose (5.5 mM) with Glutamax], PBS (including Ca^2+^ and Mg^2+^), antibiotics and heat inactivated sera (fetal bovine serum–FBS and horse serum—HS) were purchased from Gibco Life Technologies (Grand Island, NY, USA). Insulin from porcine pancreas (Sigma Aldrich Chemical Co., St. Louis, MO, USA) was dissolved in dilute acetic acid according to manufacturer recommendations and kept frozen at -20°C. Metabolic inhibitors of: PI3-K (LY294002), MAPK MEK (PD98059), GSK-3β (SB216763 and LiCl), ETC complex I (rotenone), mitochondrial ATP synthase complex (oligomycin) if necessary were dissolved in DMSO and depending on the type were kept frozen at -20°C or refrigerated at 4–8°C. All other reagents were cell culture tested, of high purity and unless otherwise stated they were purchased from Sigma-Aldrich Chemical Co. (St. Louis, MO, USA). Plastics were from Becton Dickinson (BD Biosciences, Franklin Lakes, NJ, USA), tubes for deep freezing from NunclonTM (Nunc^TM^, Roskilde, Denmark) while syringe filters were purchased from Corning-Costar Inc. (Cambridge, MA, USA).

### Muscle and Myotube Cell Cultures and Treatments

The mouse C2C12 cell line [37] was obtained from European Collection of Animal Cell Cultures (ECAAC). Cells were initially suspended in growth media (GM) containing DMEM with Glutamax supplemented with 10% (v/v) fetal bovine serum (FBS), pen:strep (Penicillin:Streptomycin solution, 50 IU/mL/50μg/mL), Gentamicin sulfate 20 μg/mL, Fungizone—Amphotericin B 1 μg/mL, and plated onto a plastic non-coated culture flasks or Petri dishes. They were cultured at 37°C in a humidified 5% CO_2_ and 95% air in incubator. After reaching 70–80% confluence, myoblasts were subcultured by trypsynization and the same volume of cell suspension was seeded onto 100 mm Petri dishes, 96-flatwell plates or multiwell 8 Chamber Culture Slides (Becton Dickinson, BD Biosciences, Franklin Lakes, NJ, USA) depending on the experimental protocol. For differentiating and differentiation states when the myoblasts reached 80% confluence, growth media were switched to differentiation medium (DM) containing DMEM with Glutamax supplemented with 2% (v/v) horse serum (HS) and the same antibiotic:antimycotic mixture. During acute and chronic studies of myogenic differentiation DM was replaced by freshly prepared media containing 2% BSA (w/v) with or without experimental factors. In the case of mitogenicity study, GM in non-confluent cells was directly replaced by 2% BSA (w/v)/DMEM with or without experimental factors. When the experimental factors were dissolved in DMSO, the equivalent volume of vehicle (0.1% v/v) was added to the control cells. DMSO-dissolved reagents were added exactly 30 min prior to the application of water-soluble reagents. Preliminary experiments were carried out with increasing concentrations of insulin and metabolic inhibitors for different time points in order to choose the best time/concentration combination to adopt in our short- and long term studies (S1 Fig). Differences in phosphorylation status (WB) and activities of particular proteins Mfn2, COX IV, GSK-3β, myogenin, FOXO1 were carried out as short term studies. Viability, mitogenicity or changes in the protein expression (WB, ICC) were investigated in the long term study. There were dose- and time-dependent cell responses suggesting that the best concentration setting in our experimental model (determined by the complete absence of cellular toxicity in MTT assay) was obtained using 10 nM of insulin (S1 Fig). Cells were removed from the culture plates using trypsin (harvesting), centrifuged in GM at 1000 rpm for 5 min, media were aspirated, and cell pellets were resuspended in GM. For the differentiating state, after 24 h in GM, cells were washed with PBS and then incubated in DM for 1 to 5 days. Media were changed every day. Myotubes were harvested either each day (Western blot) or on day 1, 3 and 5 (qRT PCR) of myogenic differentiation. Floating dead cells were removed during media change or washing with PBS and were not included in these experiments.

### Determination of Cell Viability, Mitogenicity and ATP Formation

Cell viability was based on the ability of cells grown on 96-well plates to convert soluble MTT [3-(4,5-dimethylthiazol-2-yl)-2-5-diphenyltetrazolium bromide] into an insoluble purple formazan reaction product with minor modifications to protocol described [38]. Briefly, cells were uniformly seeded in 96-well flat bottomed plates and grown in GM for 24 h. Confluent cultures were washed with PBS and then exposed to DM including (or not) experimental factor(s) for 5 successive days. Media were changed every day and relative viability (percentages of initial control value on the first day of experiment) was evaluated at each day (1, 2, 3, 4, 5). Media were removed, cells were washed with PBS, and were further incubated with MTT solution in DMEM without phenol red (w/v 5 mg/mL) for 1 h at 37°C in a humidified 5% CO_2_ and 95% air in incubator. Next, MTT solution was removed and water insoluble formazan was immediately dissolved in DMSO (100 μL per well).

Alternatively, cell viability was determined on the basis of lysosomal uptake of neutral red dye. C2C12 cells were grown in 96-well flat-bottomed plates in GM. After reaching confluence, cells were switched into post-mitotic status by incubation in DM. Wells were immersed with DM (Ctrl) and experimental media for 1, 2, 3, 4 and 5 days (percentages of initial control value on the first day of experiment). For the last 1 h of incubation, these media were replaced by 50 μL neutral red reagent (5 mg/mL in PBS, sterilized by filtration). After incubation, the medium was aspirated and cells were washed twice with PBS. Cell monolayers were allowed to dry at ambient temperature, and neutral red accumulated within lysosomes of living cells was dissolved by addition of 100 μL DMSO solution (70% v/v in dd.H_2_O).

Cell mitogenicity was determined by crystal violet (CV) assay on identical 96-well flat bottomed multiwell plates. Cells were uniformly seeded and grown in GM for 24 h. Mitogenicity was measured on day 3 of culture. Initially, cells were kept in GM for 24 h, followed by 24 h incubation with the experimental factor(s) dissolved in serum-free 2% BSA/DMEM, and finally recovered in GM for another 24 h. Cells were exposed (or not) to experimental factors during 24 h. Upon completing the experiment cells were washed with PBS and fixed with two-step bath in ice-cold methanol (70% followed by 100%, v/v, 20 min, 4°C). Cells were immersed in 0.2%, w/v crystal violet solution in dd.H_2_O with ethanol 2% (v/v) for 10 min. Subsequently, they were gently washed with dd.H_2_O, air dried and incubated with SDS solution (1%, w/v in dd.H_2_O). The absorbances for MTT, neutral red, and CV were measured at 490, 550 and 570 nm, respectively with ELISA reader type Infinite 200 PRO Tecan™ (TECAN, Austria). Relative percentages (vs. non-treated control) of viable or proliferating cells were measured by MTT conversion into purple formazan, neutral red, and quantity of CV bound to cellular DNA, respectively.

Cellular ATP was determined with ViaLight™ Plus BioAssay Kit (Lonza Rockland, Rockland, ME, USA). The kit is based upon the bioluminescent measurement of ATP that is present in all metabolically active cells. The bioluminescent method utilizes an enzyme, luciferase, which catalyses the formation of light from ATP and luciferin according to the following reaction:

$$\begin{array}{l} \;\text{Luciferase}\\ \text{ATP}+\text{Luciferin}+{\text{O}}_{2}\to \text{Oxyluciferin}+\text{AMP}+\text{PPi}+\text{LIGHT}\\ \;\text{Mg}2+ \end{array}$$

The emitted light intensity is linearly related to the ATP concentration and it was measured using the Infinite 200 PRO Tecan™ (TECAN, Austria). The assay was conducted at ambient temperature (18°C-22°C), the optimal temperature for luciferase enzymes.

### Cytochemistry and Immunocytochemistry

CC and ICC have been used to evaluate myotube index and the distribution and localization of sarcomeric myosin heavy chain (MHC) protein in myotubes, respectively. Lab-Tek 8-chamber polystyrene slides (Permanox^®^ slide, 0.8 cm^2^/well, w/Cover, Nalge Nunc International, Naperville, IL, USA) were used to culture, fix and stain the cells *in situ*. C2C12 myoblasts were propagated in Permanox slides and treated (or untreated) at appropriate times with experimental factors. After 24, 72 and 120 hours, the cells were fixed as follows: washed twice with PBS, fixed in 1% (v/v) formaldehyde for 15 minutes in room temperature, washed twice with PBS, and incubated for 10 min in RT in Triton X-100 solution (0.5% v/v in PBS). Next, cells were washed twice with PBS and immersed in 70% ethanol for 10 min in 4°C. To calculate the number of nuclei in myotubes, after fixation and permeabilization cells were immersed in bisbenzimide (Hoechst 33342, HO33342) solution in dd.H_2_O (10 μg/mL, Molecular Probes Inc., Eugene, OR, USA). After washing, mounting medium was added and slides were sealed with cover slips, while nuclei were counted in fluorescent microscope using the common DAPI filter with mercury-arc lamp. Demonstration of the presence and intracellular location of MHC by immunocytochemical detection was performed by two-step reaction. To do this, C2C12 myoblasts propagated in Permanox slides and treated (or untreated) at appropriate times with experimental factors were washed twice with PBS, fixed in 1% (v/v) formaldehyde for 15 minutes in room temperature (RT), washed twice with PBS, and incubated for 10 min in Triton X-100 solution (0.5% v/v in PBS, RT). After three washes with PBS, cells were immersed in 70% methanol for 20 min at 4°C. After subsequent double washing with PBS, cells were incubated with 1% (w/v) bovine serum albumin (PBS-BSA) + 5% (v/v) normal donkey serum (NDS) for 30 min in RT. To evaluate the expression of MHC sarcomere protein, after subsequent double washing with PBS the cells were immersed in 100 μL of primary mouse monoclonal anti-myosin IgG (1:30 v/v, clone MF20, Developmental Studies Hybridoma Bank, University of Iowa, Iowa City, IA, USA) dissolved in 1% (w/v) PBS-BSA and incubated for 60 min at 4°C. Next, the cells were washed twice in PBS and 100 μL aliquot of Alexa Fluor® 488-conjugated AffiniPure (F(ab’)_2_ fragment donkey anti-mouse IgG (1:50 v/v in 1% (w/v) BSA-PBS; Jackson ImmunoResearch Laboratories Inc., West Grove, PA, USA) was added and slides were incubated for 1 hour in the dark at 4°C. No primary mouse monoclonal anti-myosin IgG but solely Alexa Fluor® 488-conjugated AffiniPure (F(ab’)_2_ fragment donkey anti-mouse IgG were used as the isotype negative control. The samples were counterstained with HO33342 solution in dd.H_2_O (10 μg/mL, Molecular Probes Inc., Eugene, OR, USA). After a subsequent double wash with PBS and aspiration, sufficient volume of mounting medium (Mowiol, Calbiochem-Novabiochem Co. La Jolla, CA, USA) was applied prior to coverslips placement on slides. Cells were observed, analyzed and photographed under fluorescence microscope (Olympus BX-60, Olympus Poland, Warsaw, Poland).

### Myotube Formation and Fusion Index

Multinuclear (>3 myonuclei per cell) myotubes were identified using fluorescence microscope (Olympus BX-60, Olympus Poland, Warsaw, Poland) in 10 randomly chosen microscopic fields with at least 100 nuclei (S4 Fig). HO33342 stained cells were compared with the same field by contrast phase highlighting. Cells were considered myotubes if they contained at least three nuclei within their cytoplasm. Numbers of nuclei within myotubes in 10 randomly selected fields were counted on each replicate well, and the average number of all nuclei per field was calculated for each well. Three independent experiments were performed. The fusion index was defined as the relation between the number of nuclei within myotubes (>3) and the average of the whole number of nuclei multiplied by 100% (S4 Fig).

### Antibodies, Immunoblotting, Immunoprecipitation, Transcriptional Activity of FOXO1, and Microscopic Imaging

For analysis of protein expression, 30 μg of protein isolated from whole-cell lysates and wide-range molecular weight standards (Precision Plus Protein™ Kaleidoscope™, Bio-Rad Polska, Warsaw, Poland) were electrophoresed on a 7.5, 10 or 12% acrylamide SDS-PAGE gels and immunoblotted onto polyvinylidene difluoride Immun-Blot® PVDF Membranes (Bio-Rad Polska, Warsaw, Poland). The membranes were blocked for 1 h in room temperature either with 5% nonfat dry milk (NFDM) w/v in TBST (NaCl 137 mM, KCl 2.7 mM, Tris base 19 mM) or in 5% BSA w/v in TBST (depending on the antibody used). Cells were cultured with or without experimental factors indicated in figure legends, harvested, washed, and lysed with RIPA lysis buffer (1x PBS, 10 mL/l Igepal CA-630, 5 g/L sodium deoxycholate, 1 g/L SDS) supplemented with 0.4 mM PMSF, 10 μg/mL of aprotinin and 10 μg/mL of sodium orthovanadate was added. To lyse the cell pellets, cells were broke up by repetitive triturating with the syringe with attached needle (21G, 0.8 mm diameter). Cell suspension was then left on ice (4°C) for 30 min, and centrifuged for another 5 min (4°C, 8,000xg). To separate cytoplasmic and nuclear fractions, cells were washed, and after centrifugation cell pellets were resuspended in 400 μL of ice-cold buffer (10 mM HEPES pH 7.9; 10 mM KCl; 0.1 mM EDTA; 0.1 mM EGTA; 1 mM DTT; 0.5 mM PMSF), and incubated on ice for 15 min. Then 25 μL of a 10% solution of Igepal CA-630 was added. After centrifugation, supernatants containing cytoplasm were transferred to fresh tubes and were stored at -80°C. Nuclear pellets were resuspended in 200 μL RIPA buffer (1xPBS; 1% Igepal CA-630; 0.5% sodium deoxycholate; 0.1% SDS; aprotinin (available as a liquid from Sigma-Aldrich Chemical Co.; 30 μL added to 1mL of buffer; 1 mM sodium orthovanadate) and were passed through a 21G (0.8 mm diameter) needle. PMSF (0.1 mg/mL) was added and cells were incubated 30 min on ice. After centrifugation, cytoplasmic and nuclear lysates were stored at -80°C until analysis. For protein quantification in the whole-cell lysates a protein-dye-binding method [39] with commercial reagent was used (Bio-Rad Laboratories, Hercules, CA, USA).

Antibodies against listed proteins were used: actin, myogenin, Mfn2, MyoD, AKT-1, P-S473-AKT-1, ERK1/2, P-T202/Y204-ERK1/2, GSK-3β, P-S9-GSK-3β (Santa Cruz Biotechnologies, Santa Cruz, CA, USA), P-Y216-GSK-3β (Becton Dickinson, Franklin Lakes, NJ, USA), cytochrome *c* oxidase subunit IV (Molecular Probes, Eugene, OR, USA). Working antibody concentrations (from 1:200 to 1:2000) varied depending on the protein detected, and were applied according to the manufacturer’s recommendation. After detection with antibodies which distinguished phosphorylated proteins, the same blot was reprobed with antibodies against the non-phosphorylated form of the same protein in order to verify that equal amounts of protein were always loaded and to identify the proteins. Secondary polyclonal antibodies (Santa Cruz Biotechnology, Santa Cruz, CA, USA) raised against respective species and conjugated to horseradish peroxidase were used for detection, followed by enhanced chemiluminescence assay (Amersham International, Aylesbury, U.K.). After exposure, and processing the films were scanned and analyzed using Kodak EDAS 290/Kodak 1D 3.5 system.

For immunoprecipitation at particular time points the cells were scraped from substratum in 0.5 mL RIPA buffer and after repetitive triturating with the 21G (0.8 mm diameter) needle, 0.1 mg/mL PMSF was added and cells were incubated 30 min on ice. After centrifugation the protein concentration was determined by a protein-dye-binding method as previously described. Cell lysates containing 900 μg of protein were incubated overnight at 4°C with 1.5 μg rabbit polyclonal anti-GSK-3β IgG and for additional 3 h were incubated with 30 μL protein A/G bead slurry (Santa Cruz, CA). Beads were washed 4 times with ice-cold RIPA buffer, boiled with sample buffer (2xLaemmli buffer, Sigma-Aldrich Chemical Co., St. Louis, MO, USA) and separated by 10% SDS/PAGE. After electrotransfer, the membranes were immunostained for Mfn2 protein by standard Western blot procedure.

FOXO-1 transcriptional activity was quantified with TransAM^®^ Kits (Rixensart, Belgium). These are sensitive, non-radioactive transcription factor ELISA kits that facilitate the study of transcription factor activation in mammalian tissue and cell extracts. The active form of FOXO1 contained in nuclear extracts was specifically bound to the immobilized oligonucleotide containing forkhead box protein 1 transcription factor consensus binding site (5’-TTGTTTAC-3’). The primary antibody used to detect FOXO recognized only the transcription factor that is accessible only when FOXO1 was activated and bound to its target DNA. Finally, an HRP-conjugated secondary antibody provided a sensitive colorimetric readout that was quantified by spectrophotometry (λ = 450 nm) with Infinite 200 PRO Tecan™ (TECAN, Austria).

Morphological changes and cell survival were monitored under an inverted phase-contrast microscope (Olympus CK40, model: ICD703WP). The formation of myotubes was monitored by obtaining photomicrographs using digital camera (CCD Color Camera, Hamburg, Germany).

### RNA Isolation and Quantitative Real-Time Reverse-Transcription-Polymerase Chain Reaction (qRT-PCR)

Gene activities were measured in the 1^st^, 3^rd^, and 5^th^ day of myogenesis. Total RNA was extracted from myotubes with the Total RNA Maxi kit (A&A Biotechnology, Gdynia, Poland) according to the manufacturer’s protocol. Quality check was performed with NanoDrop to assess sample concentration and purity, whereas http://www.genome.duke.edu/cores/microarray/services/rna-qc/documents/Globin reduction protocol A method.pdfAgilent Bioanalyzer was used to assess RNA integrity. RNA was frozen at -76°C before the performing the reverse transcription reaction (RT-PCR). Then, 500 μg of total RNA was purified on silica gel, and reverse-transcribed with Enhanced Avian HS RT-PCR-100 Kit (Sigma-Aldrich, Taufkirchen, Germany). Reaction mixture was based on anchored Oligo(dT)_23_ (0.5 μg/μL in water for PCR). RT-PCR was carried out using Mastercycler Personal (Eppendorf, New York, NY, USA). First, the RNA samples (200 ng/mL) were incubated for 10 min at 70°C. Then water for PCR, 10XAMV-RT buffer [500 mM Tris-HCl, pH 8.3, 400 mM KCl, 80 mM MgCl_2_, 10 mM DTT, RNAse inhibitor (20 U/μL), and reverse transcriptase AMV (20 U/μL w 200 mM KH_2_PO_4_, pH 7.2, 2 mM DTT, 0.2% (v/v) triton, 50% (v/v) glycerol] were added at 4°C. The samples were subjected to RT-PCR for 50 min at 48°C. After completing the reaction the concentration of newly synthesized cDNA was measured in the NanoDrop 1000 (NanoDrop® Technologies, Wilmington, NC, USA) at λ = 230 nm. cDNA was kept frozen at -76°C until further analyses. To perform real time PCR reaction, cDNA was combined with 25 μM of each primer (sense and antisense) and SYBR green (LightCycler® FastStart DNA Master SYBR Green I and LightCycler®Control Kit DNA; Roche Diagnostics, Warsaw, Poland). The qRT PCR measurements of individual cDNAs were performed in triplicates using SYBR green dye to measure duplex DNA formation with the LightCycler (Roche Diagnostics, Warsaw, Poland). The results were analyzed with LightCycler3 Front Screen. *18S rRNA* was used as a reference gene. The sequences of the primers sets used are shown in the attached Table 1 (GenBank). The relative mRNA levels of the target genes were determined using the relative standard curve.

**Table 1:** Primers and their sequences used to identify genes in quantitative real-time reverse-transcription-polymerase chain reaction (qRT-PCR).

Gene	Primers and their sequences	MgCl_2_ (mM)	Product (bp)
*Mfn2*	F: 5' -GCCAGCTTCCTTGAAGACAC-3'R: 5' -GCAGAACTTTGTCCCAGAGC-3'	2	208
*MtSSB*	F: 5`-CAAATGAGATGTGGCGATCA-3`R: 5`-GTCCACTTTCCCTTCCACAA-3`	1.5	164
*mtTFA*	F: 5'-CCAAAAAGACCTCGTTCAGC-3'R: 5 -CTTCAGCCATCTGCTCTTCC-3'	1.5	211
*Cox-1*	F: 5’-GCCTTTCAGGAATACCACGA-3’R: 5’-AGGTTGGTTCCTCGAATGTG -3’	3	234
*18S rRNA*	F: 5’-GGAGAGCGGGTAAGAGAGGT-3’	2	235
	R: 5’-CAGGACTAGGCGGAACAGAG-3’		

PCR conditions were as follows: denaturation for each cycle 95°C (35 cycles, 10 sec for each cycle); annealing for *Mfn2*, *MtSSB*, *mtTFA* 58°C (35 cycles, 0–10 sec for each cycle); and for *Cox-1*, and *18S rRNA* 56°C (35 cycles, 0–10 sec for each cycle); elongation 72°C (35 cycles, 4–5 sec for each cycle).

### Statistical Analysis

Each experiment was repeated at least three times. Statistical analyses were performed using one-way analysis of variance (ANOVA) followed by Kruskal-Willis, Tukey’s, Newman-Keuls’a or Benferroni multiple comparison post-tests and with *P*-value being adjusted for multiple comparisons. If necessary, the selection of particular post-hoc test (Newman–Keuls, Tukey or Benferroni) was performed after the same critical difference for the first comparison (Kruskal-Willis) was tested. Regression analysis (4^th^ order polynomial) was carried out to draw appropriate dose-response or time-course curves. *P* values of less than 0.05 were considered statistically significant. Statistical differences from non-treated control cells were indicated by asterisks (* for *p*<0.05; ** for *p*<0.01; *** for *p*<0.001), whereas statistical differences between the treatments and untreated control cells were ticked with different lower case letters (bar charts). All the results (if not otherwise stated) are presented as mean ± SE. Statistical analyses were performed using GraphPad Prism^TM^ version 5.0 software (GraphPad Software Inc., San Diego, CA, USA).

## Results

### Effects of Some Metabolic Inhibitors on Insulin-Mediated Myogenesis from C2C12 Myoblasts Suggest that Both PI3-K/AKT, Mitochondrial Electron Transport Chain, and ATP Synthase Activities Are Essential for Muscle Cell Viability

**Fig 1:**
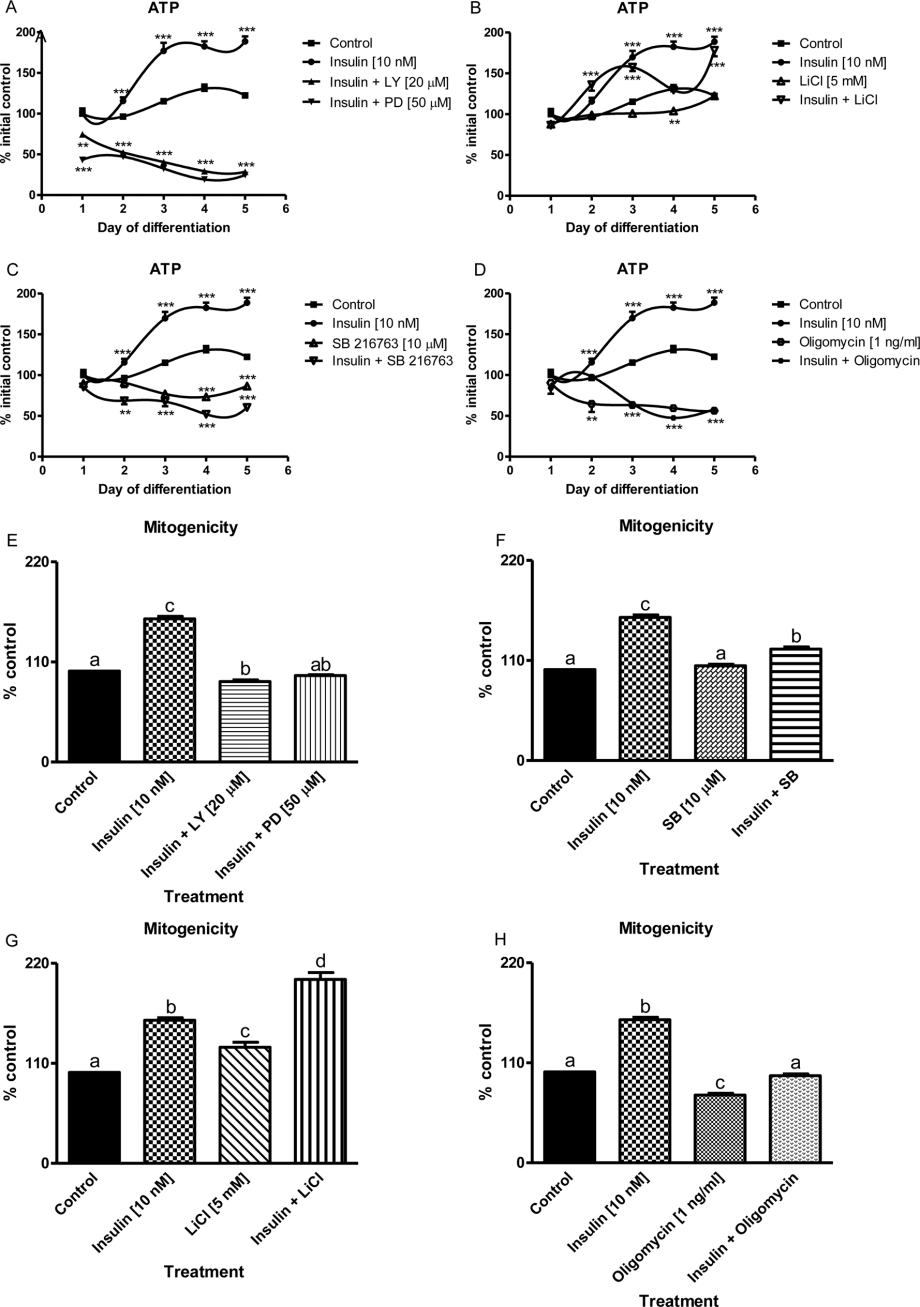
The influence of metabolic inhibitors on long-term insulin-dependent biochemical effects. Long term effects of insulin (10 nM) alone or given together with selected metabolic inhibitors (at indicated concentrations) on cellular ATP synthesis (a, b, c, d) during 5 subsequent days of myogenic differentiation (regression analysis 4^th^ order polynomial). Statistical differences from non-treated control cells are indicated by asterisks (* for *p*<0.05; ** for *p*<0.01; *** for *p*<0.001). Mitogenicity (DNA synthesis, % control) was measured with crystal violet assay at the 3^rd^ day of muscle differentiation (e, f, g, h). The results are indicative of three independent experiments performed in eight replicates. Statistical differences between the treatments and untreated control cells are ticked with different lower case letters.

**Fig 2:**
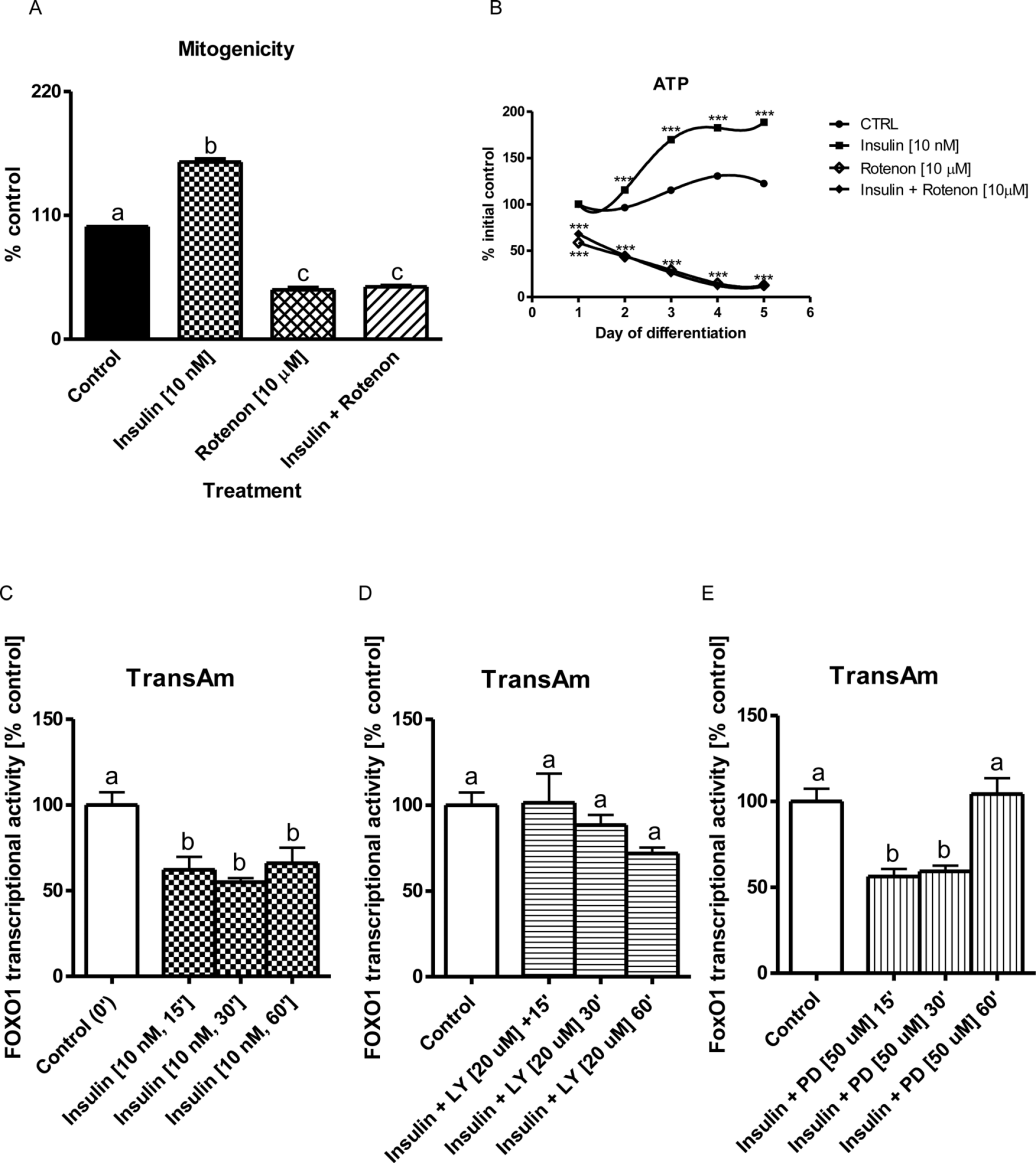
Insulin-dependent mitogenicity and FOXO1 transcriptional activity. Mitogenicity (DNA synthesis) measured with crystal violet assay at the 3^rd^ day of muscle differentiation (a, % control) and cellular ATP synthesis (b, regression analysis 4^th^ order polynomial) during 5 subsequent days of myogenic differentiation. The results are indicative of three independent experiments performed in eight replicates. FOXO1 transcriptional activity in the 3^rd^ day of myogenesis (c, d, e, % control). The results are indicative of three independent experiments performed in triplicates. Statistical differences from non-treated control cells are indicated by asterisks (* for *p*<0.05; ** for *p*<0.01; *** for *p*<0.001, regression analyses), whereas statistical differences between the treatments and untreated control cells are ticked with different lower case letters (bar charts).

An initial rise followed by a plateau phase and a moderate fall in both cell viability (MTT), lysosomal integrity (NR) and intracellular ATP levels were observed in non-treated C2C12 muscle cell cultures (S1 Fig, Fig 1). Insulin (10 nM) markedly recovered these indices except at the day 5 of myogenesis (S1 Fig, *p*<0.001). Inhibitors of PI3-K (LY294002, 20 μM), MAPK MEK (PD98059, 50 μM), cytochrome *c* oxidase/complex IV (rotenone, 10 μM), ATP synthase (oligomycin, 1 ng/mL), GSK-3β (SB216763, 10 μM) inhibited both respiration, lysosome integrity and ATP synthesis in C2C12 myotubes compared to non-treated cells (S1 Fig, Fig 1, Fig 2A and 2B, *p*<0.001). Thus, administration of the aforementioned inhibitors abrogated insulin-stimulated muscle cell survival. In contrast to SB216763 (S1 Fig, Fig 1C and 1H), another GSK-3β inhibitor (LiCl, 5 mM) when given alone did not affect muscle cell viability but it markedly facilitated insulin-dependent muscle cell viability (S1 Fig, 1b and 1g, *p*<0.001).

### Insulin Stimulates Whereas Some Metabolic Inhibitors Retard Insulin-Dependent Mitogenicity and Myogenesis in C2C12 Muscle Cells

Insulin (10 nM) was apparently mitogenic at day 3 of myogenesis as it elevated considerably intracellular DNA level with regard to non-treated muscle cells (Fig 1E–1H, *p*<0.01). Inhibitors of PI3-K (LY294002, 20 μM), MAPK MEK (PD98059, 50 μM), cytochrome *c* oxidase/complex IV (rotenone, 10 μM) and ATP synthase (oligomycin, 1 ng/mL) diminished mitogenicity compared to control conditions (Figs 1E–1H and 2A, *p*<0.05). Thus, all aforementioned inhibitors prevented insulin-mediated mitogenicity. In contrast, LiCl inhibitor (5 mM) even stimulated muscle cell viability and markedly augmented insulin-stimulated muscle cell mitogenicity (S1 Fig, Fig 1B, Fig 1G, *p*<0.001). Astonishingly, if given alone, no effect of the GSK-3β inhibitor SB216763 (10 μM) was found but it markedly inhibited insulin-stimulated DNA synthesis (Fig 1F, *p*<0.05).

**Fig 3:**
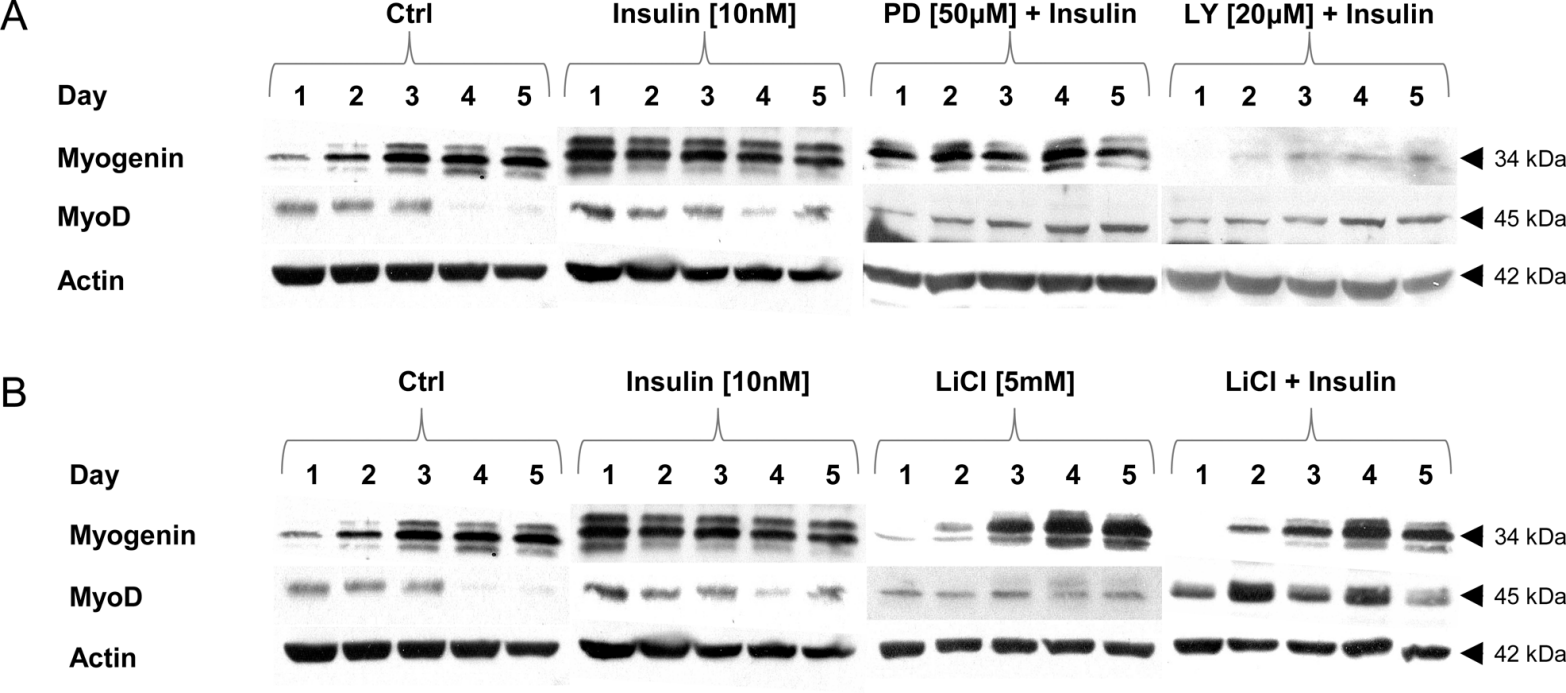
The effects of metabolic inhibitors on insulin-dependent expression of myogenic markers. Long term effects of insulin (10 nM) given alone, or with PI3-K (LY294002) or MEK (PD98059) inhibitors (at indicated concentrations). Protein expression of MyoD and myogenin was analyzed during 5 subsequent days of myogenic differentiation. Actin was used as loading control. The results are indicative of three independent experiments.

C2C12 muscle cells kept in DM, spontaneously differentiate into myotubes within 5 days. Expression of MyoD was elevated at day 1, 2 and 3 but it was almost undetectable at day 4 and 5 (Fig 3A). Insulin extended MyoD expression phase to day 4 and 5 (Fig 3A) irrespective to metabolic inhibitors added (LY294002, 20 μM; PD98059, 50 μM; LiCl, 5 mM). Insulin administration (10 nM) substantially stimulated myogenesis demonstrating both earlier (1^st^ day) and higher myogenin protein expression when compared to untreated muscle cells (Fig 3A, S2 Fig). Concomitant use of PI3-K inhibitor LY294002 (20 μM) and insulin prevented myogenin as well as myotube formation but it did not affect MyoD expression levels (Fig 3A, S2 Fig). Addition of MAPK MEK inhibitor PD98059 (50 μM) (Fig 3A, S2 Fig) or LiCl (5 mM) (Fig 3B, S3 Fig) neither affected expressions of MyoD or myogenin nor insulin-mediated myogenesis. These observations were validated by measurement of fusion index and intracellular MHC expression in IC and ICC approach, respectively (S4 and S6 Figs).

### Insulin Inhibits Transcriptional Activity of FOXO1 in C2C12 myotubes. It Is Reversed by LY294002 More Rapidly than by PD98059 Administration

Transcriptional activity was evaluated using the TransAM^TM^ method. As shown on Fig 2C, when C2C12 myotubes were exposed to insulin (10 nM), the transcriptional activity of FOXO1 was markedly reduced by approximately 40% at the 15^th^, 30^th^ and 60^th^ minute of treatment (*p*<0.001 compared to control). In contrast to insulin given alone, after simultaneous use of PI3-K inhibitor LY294002 (20 μM) but not PD98059 (50 μM), FOXO1 transcriptional activity was almost immediately restored to control level (Fig 2D, *p*>0.05).

### Insulin Stimulates *Mfn2*, *Cox-1*, *MtSSB* and *mtTFA* Gene Activities. Insulin-Dependent *Mfn2*, *Cox-1*, *MtSSB* Activation Is Neither Modulated by PI3-K, MAPK MEK nor GSK-3β Inhibitors. In Contrast, GSK-3β Is Important for Insulin-Mediated *mtTFA* Gene Activation as the Latter Dropped after Concomitant LiCl or SB216763 Co-Treatment

**Fig 4:**
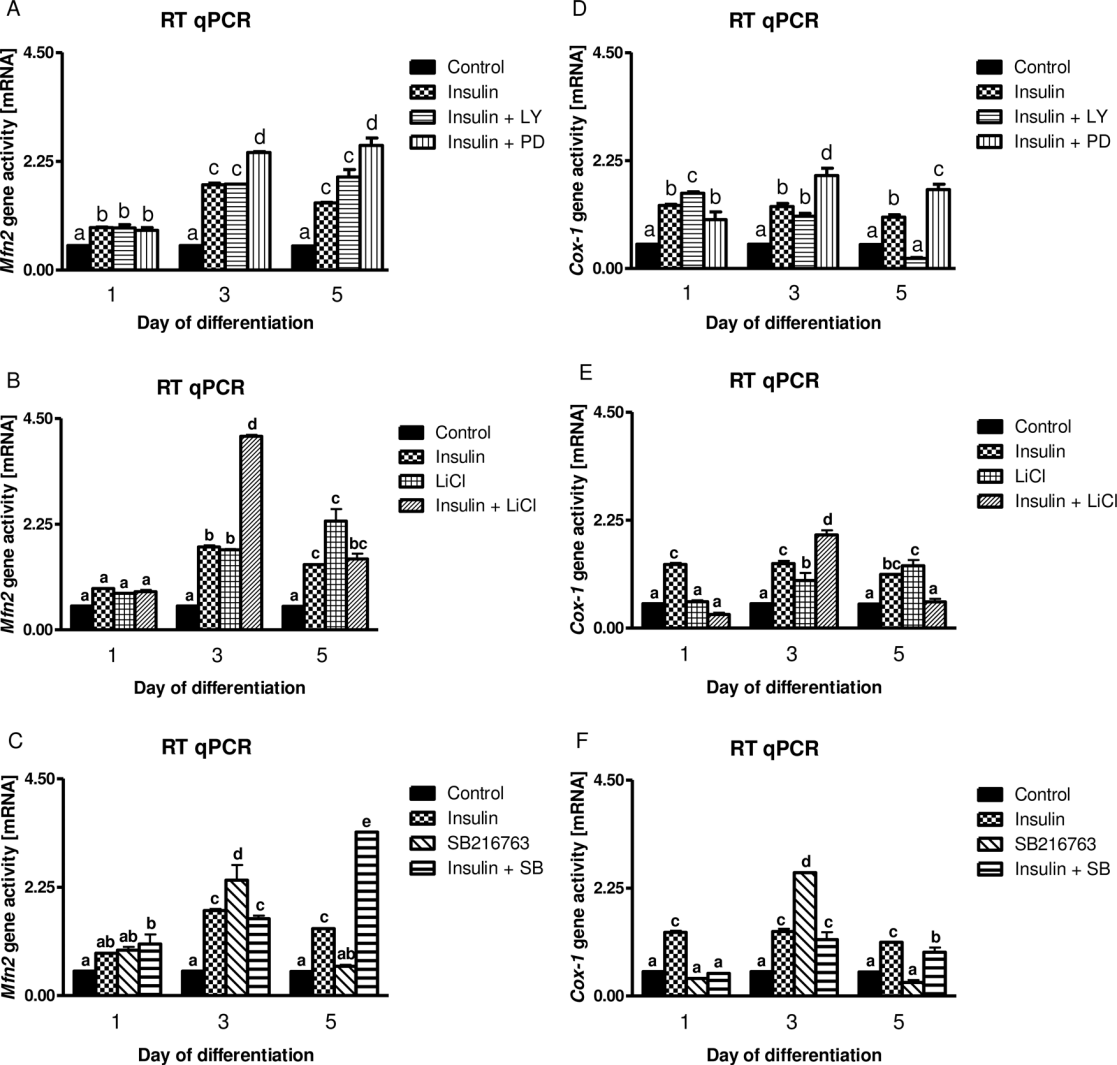
The influence of metabolic inhibitors on long-term insulin-dependent control of *Mfn2* and *Cox-1* genes. Long term effects of insulin (10 nM) given alone (chessboard) or with metabolic inhibitors (checkered and slashed bars) at indicated concentrations (20 μM for LY294002, 50 μM for PD98059, 5 mM for LiCl, and 10 μM for SB216763) on the *Mfn2* and *Cox-1* gene activities in the 1^st^, 3^rd^, and 5^th^ day of myogenesis. Fold increase was calculated according to the formula described in Materials and Methods section. Statistical differences between the treatments and untreated control cells are ticked with different lower case letters.

**Fig 5:**
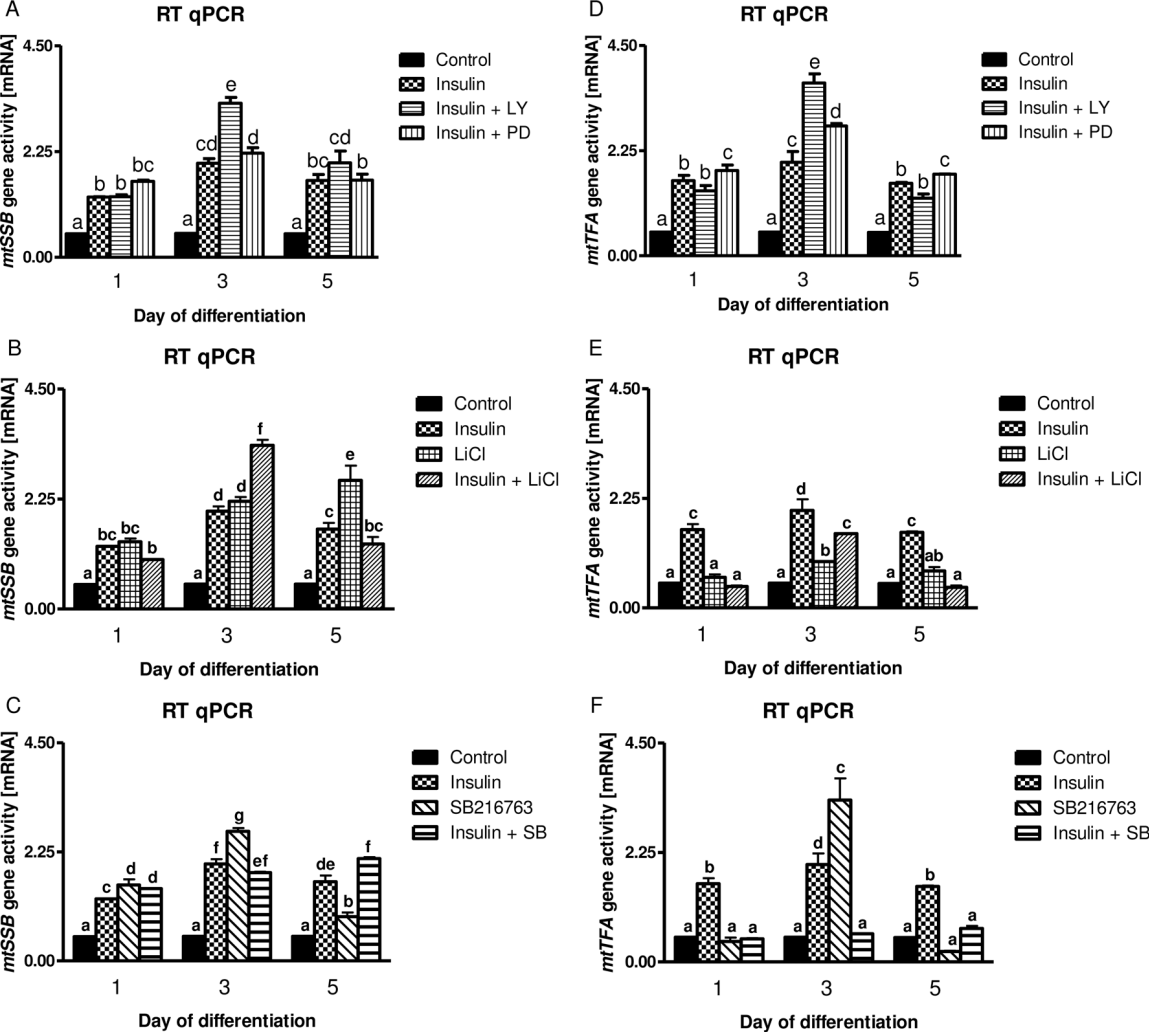
The influence of metabolic inhibitors on long-term insulin-dependent control of *MtSSB* and *mtTFA* genes. Long term effects of insulin (10 nM) given alone (chessboard) or given together with metabolic inhibitors (checkered and slashed bars) at indicated concentrations (20 μM for LY294002, 50 μM for PD98059, 5 mM for LiCl, and 10 μM for SB216763) on the *MtSSB* and *mtTFA* gene activities in the 1^st^, 3^rd^, and 5^th^ day of myogenesis. Fold increase was calculated according to the formula described in Materials and Methods section. Statistical differences between the treatments and untreated control cells are ticked with different lower case letters.

Insulin stimulated *Mfn2*, *Cox-1*, *MtSSB* and *mtTFA* gene activities at the 1^st^, 3^rd^, and 5^th^ day of myogenesis (Figs 4 and 5, *p*<0.01). Administration of LY294002 (PI3-K inhibitor) or PD98059 (MAPK MEK inhibitor) or LiCl/SB216763 (GSK-3β inhibitors) could not reverse insulin-dependent stimulation of *Mfn2*, *Cox-1* and *MtSSB* gene activities (Figs 4 and 5, *p*>0.05). Interestingly, PD98059 even enhanced insulin effect with respect to *Mfn2*, *Cox-1* and *mtTFA* but not that of *MtSSB* gene activity (Figs 4 and 5, *p*<0.01). Similarly to LiCl (5 mM), another inhibitor of GSK-3β (SB216763, 10 μM) attenuated insulin-mediated activation of *mtTFA* (Figs 4 and 5, *p*<0.01).

### Expression of Myogenin Is Inhibited whereas MyoD Input Is Markedly Delayed after Concomitant Administration of PI3-K Inhibitor (LY294002). LiCl Amplifies Insulin-Dependent MyoD Expression

Immunoblotting revealed that in untreated muscle cells the muscle regulatory factors (MRFs) were typically expressed in myogenesis induced by switch from GM to DM. Initially, MyoD expression was elevated (day 1–3), while myogenin expression rose progressively from day 2 to 5 (Fig 3). Insulin noticeably increased myogenin expression but this effect was inhibited by simultaneous treatment with PI3-K inhibitor (LY294002) (Fig 3A). Administration of MAPK MEK inhibitor (PD98059) did not affect insulin–mediated changes in MRFs expression while LiCl amplified the MyoD input (Fig 3A and 3B).

### AKT/PKB Controls S9 and Y216 Phosphorylation of GSK-3β. Changes in Active Form of AKT (P-S473) Are Associated with Increased ERK1/2 and P-T202/Y204 ERK1/2 Expression Levels

**Fig 6:**
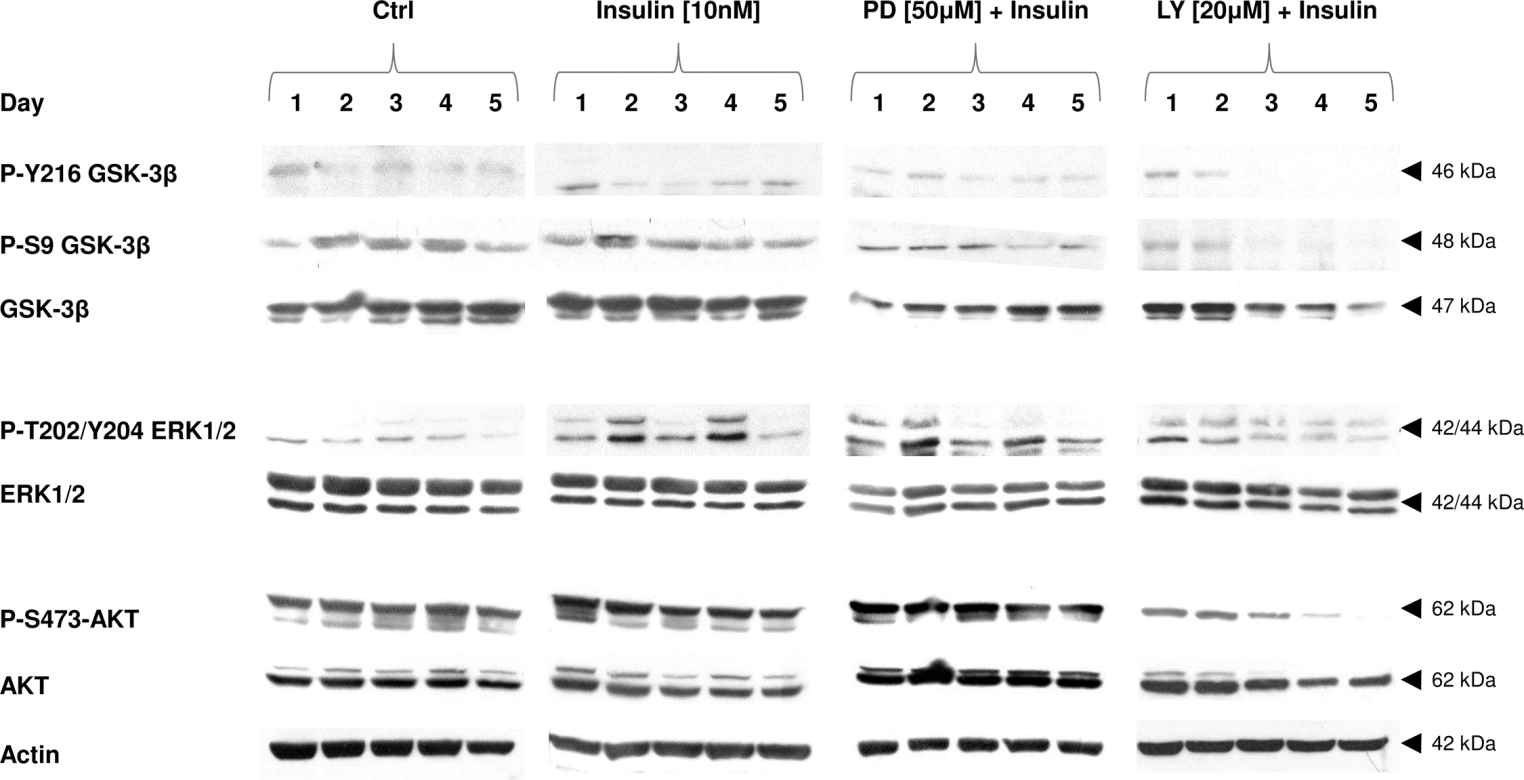
The effects of metabolic inhibitors on insulin-dependent cell signaling. Long term effects of insulin (10 nM) given alone, or with PI3-K (LY294002) or MEK (PD98059) inhibitors (at concentrations indicated). Protein expression of P-Y216 GSK-3β, P-S9 GSK-3β, GSK-3β, P-T202/Y204 ERK1/2, ERK1/2, P-S473-AKT, and AKT was analyzed during 5 subsequent days of myogenic differentiation. Actin was used as loading control. The results are indicative of three independent experiments.

GSK-3β is an important substrate for AKT/PKB. The phosphorylation status of the GSK-3β is widely used to assess insulin activity in target cells. In untreated C2C12 muscle cells GSK-3β phosphorylation at S9 and Y216 was negligible (Fig 6). P-T202/Y204 ERK1/2 expression levels raised after insulin treatment whereas AKT-dependent GSK-3β phosphorylation at S9 was rapidly ceased by concomitant LY294002 but not PD98059 co-treatment (Fig 6).

### Insulin Increases Mfn-2 and Nucleus Encoded Cytochrome *c* Oxidase Subunit IV (COX IV) Expression Levels. Insulin Effect Is Repressed after Concomitant PI3-K Inhibitor (LY294002) Administration. In Turn, Insulin Effect Is Further Amplified by LiCl but not SB216763 GSK-3β Inhibitor

**Fig 7:**
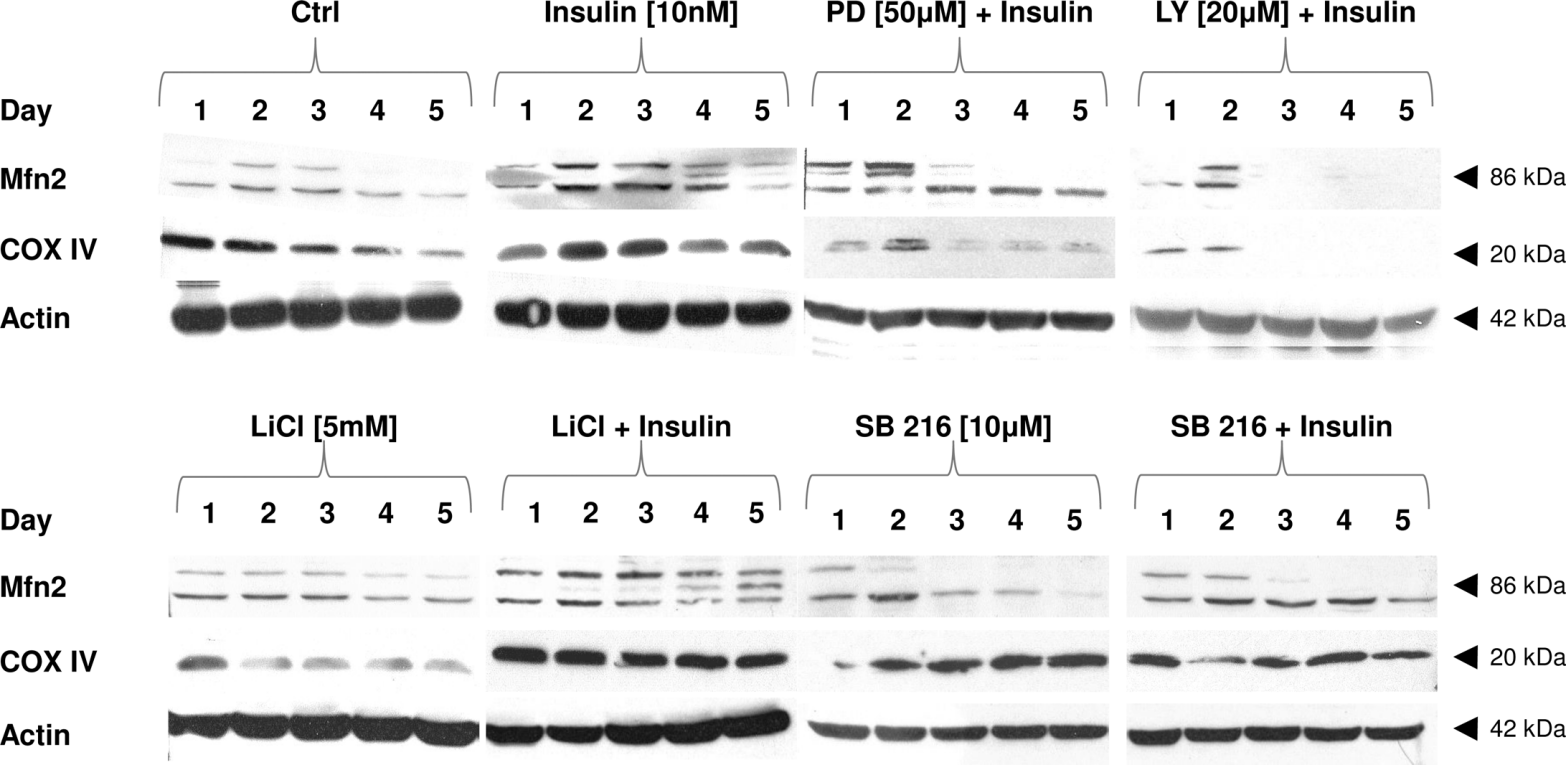
The effects of metabolic inhibitors on insulin-dependent expression of Mfn2 and COX IV proteins. Long term effects of insulin (10 nM) given alone, or with PI3-K (LY294002) or MEK (PD98059) inhibitors (at indicated concentrations). Protein expression of Mfn2 and COX IV was analyzed during 5 subsequent days of myogenic differentiation. Actin was used as loading control. The results are indicative of three independent experiments.

Mitochondrial cytochrome *c* oxidase activity is the major regulatory site for oxidative phosphorylation. Consequently, COX activity and its expression are essential for utilization of electrons liable for the maintenance of proton gradient essential for ATP synthesis [40]. In turn, Mfn-2 protein is known to control fusion of mitochondria and indirectly facilitates delivery of mitochondrial energy stores. Insulin-induced myogenesis was accompanied by augmented expression of nuclear-encoded subunit IV of COX and Mfn-2 proteins (Fig 7). Insulin-mediated effect was inhibited by PI3-K inhibitor LY294002 (20 μM), but not MAPK MEK inhibitor PD98059 (50 μM). None of the GSK-3β inhibitors given alone (LiCl or SB216763) affected referred COX IV and Mfn-2 protein expression levels (Fig 7). Interestingly, if administered with insulin LiCl but not SB216763 GSK-3β inhibitor enhanced COX IV and Mfn-2 protein expression.

**Fig 8:**
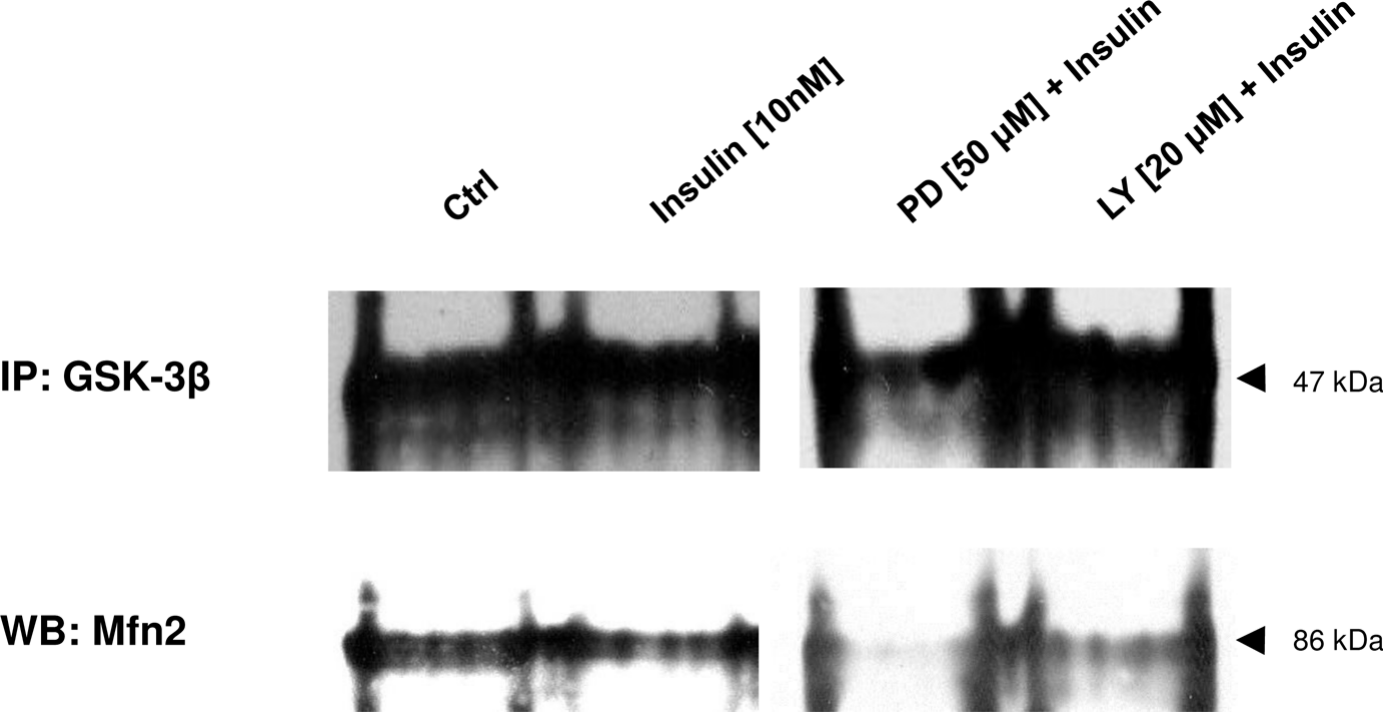
Mfn2 interaction with GSK-3β. Immunoprecipitation of GSK-3β shows the effect of insulin given alone, or with PI3-K (LY294002) or MEK (PD98059) inhibitors (at indicated concentrations). Immunoreactive Mfn2 in the precipitates upon insulin and/or co-treatment in the 3^rd^ day of myogenesis. IgG was used as equal input control. The results are indicative of three independent experiments.

To evaluate whether physical interaction occurs between Mfn-2 and GSK-3β the immunoprecipitation study was employed as described in Materials and Methods. It is apparent from Fig 8, that GSK-3β was bound to Mfn-2 protein in untreated muscle cells at the time of extensive myotube formation (3^rd^ day of myogenesis). Quantity of Mfn-2 in the complex was neither affected by insulin or insulin with LY294002 (Fig 8). It was however, notably reduced when insulin was given together with MAPK MEK inhibitor PD98059.

### Either Insulin or LiCl, and Insulin and LiCl Given Together Facilitate Myoblast Fusion Calculated by Fusion Index. LY294002 Totally Abrogated Insulin Effect

To asses how selected metabolic inhibitors affect myoblasts fusion and fusion controlled by insulin administration we calculated fusion index. As shown on bar chart (S4 Fig) insulin and LiCl stimulated myoblasts fusion and this effect was potentiated by LiCl at day 3 and 5. These data are consistent with the observation that LiCl as GSK-3β inhibitor enhanced insulin-dependent COX IV and Mfn-2 protein expression. Both proteins are essential for myogenesis [4, 9]. In turn, complete lack of fusion in cultures treated with insulin and LY294002 are in agreement with earlier notice that insulin-dependent myogenesis is under PI3-K/AKT control (Fig 3A, S2 Fig). PD98059 addition did not affect insulin-dependent myogenesis.

### Cytoimmunofluorescence. The Influence of Metabolic Inhibitors on Long-Term Insulin-Dependent Myosin Sarcomere Expression

Myotube and muscle fiber formation are associated with induction and progressive rise in myosin sarcomere expression levels. We detected myosin sarcomere intracellular location by fluorescence microscopy aimed to detect green light emitted by Alexa Fluor® 488 after excitation. As shown on supplemental data (S5 and S6 Figs) C2C12 myoblasts that underwent fusion expressed MHC. The largest myotubes with extensive cytoimmunofluorescence were observed in cultures treated with insulin [10 nM], or LiCl [5 mM], or when insulin was administered with LiCl. No detectable green light from myosin sarcomere protein was noticed in LY294002 treated cultures.

## Discussion

Myogenic differentiation is a convenient model to study the pro- or anti-catabolic activities of cytokines. Skeletal muscle differentiation is featured by cell cycle arrest, followed by myoblast alignment, elongation, and fusion into multinucleated myotubes. Insulin and IGF-1 are generally known to stimulate both proliferation and differentiation of myoblasts [41–42]. A rapid and transient calcium increase from intracellular stores was shown for IGF-1-stimulated the phospholipase C gamma (PLC γ)/inositol 1,4,5-triphosphate (IP_3_)-dependent signaling pathways. Moreover, IGF-1 regulated myostatin transcription through the activation of the NFAT transcription factor in an IP_3_/calcium-dependent manner [43]. As PIK-3 plays fundamental role in this response (LY294002 and dominant negative p110 gamma completely block skeletal muscle differentiation) the LY294002 was chosen in our study to control insulin action.

In our previous paper [44] we showed that cytokine-induced muscle atrophy is mediated by NF-κB and STAT-1α which both cause upregulation of muscle-specific atrogenes (atrogin-1 and MuRF1) that can’t be prevented by insulin. Dehoux et al. [45] also observed that IGF-1 could not overcome the catabolic effect induced by proinflammatory cytokines despite activation of Akt/FOXO and GSK-β pathways and inhibition of atrogin-1 mRNA. In the present study we asked if FOXO1 and/or GSK-3β are targets in insulin-driven muscle growth and mitochondrial activity. C2C12 cell culture model was used to concomitantly unravel the role of insulin-dependent signaling circuit in mitochondriogenesis of skeletal muscle cells.

We observed that insulin (10 nM) stimulated vital functions of myoblasts and myotubes (viability, mitogenicity, lysosomal integrity, ATP formation, S1 Fig, Fig 1A–1J and Fig 1A–1D, *p*<0.01). Rise of all the aforementioned parameters was attenuated with addition of either specific inhibitor (LY 294002 for PI3-K; PD98059 for MEK, SB216763 for GSK-3β, rotenone for cytochrome *c* oxidase, and oligomycin for ATP synthase, *p*<0.001). In contrast, when LiCl was added together with insulin to block GSK-3β activity, the analyzed parameters were increased even more than insulin did it alone (*p*<0.05). Such results are in accord with data published [29] where the authors reported that GSK-3β is pivotal for skeletal muscle atrophy when examined in C2C12 myotubes. Similarly, in our study the inhibition of GSK-3β phosphorylation at Ser^9^ by LiCl (5 mM) further increased DNA synthesis stimulated by insulin (Fig 1G). In contrast to insulin and/or LiCl, mitogenicity was markedly inhibited by LY294002, SB216763, oligomycin, and rotenone even in the presence of insulin (Fig 1E and 1F, and 1H, Fig 2A, *p*<0.001). Summing up, insulin-dependent mitogenicity and myogenesis are mediated by AKT signaling through inhibition of GSK-3β. Similar conclusions were drawn from studies on muscle biopsies obtained from human beings before and after a period of resistance training (hypertrophy) and again after a period of de-training (atrophy) [46]. In our study, we used pharmacological inhibition techniques as to measure the active phosphorylated protein content of AKT and its downstream target GSK-3β and atrophy controlling transcription factor FOXO1. By contrast to LY294002 (20 μM), the reversal of insulin-mediated repression of FOXO1 activity was markedly delayed after administration of MAPK MEK inhibitor PD98059 (50 μM (Fig 2E, *p*>0.05 compared to control).

Some muscle regulatory factors (MyoD and myogenin) were exploited to supervise myotube formation. We observed that protein level of MyoD and myogenin were differently controlled by PI3-K (LY294002) and MEK (PD98059) inhibitors. MyoD protein content was detectable on the 3^rd^ day of myogenesis and although it decreased in subsequent days it was extended upon insulin treatment to day 5 (Fig 3A). Neither inhibitor (PI3-K nor MEK) could abbreviate lengthened period of MyoD protein expression brought about by insulin (Fig 3A). This observation suggests that MyoD is hardly regulated by PI3-K or MEK but it is probably upon other control mechanism(s). Myotubes were formed extensively on the day 3 which peaked on day 5, moreover, insulin significantly facilitated muscle cell fusion and LiCl (GSK-3β inhibitor) additionally amplified insulin effect (S4 Fig). Insulin-dependent myotube formation was totally inhibited by LY294002 administration. Consistently, myosin heavy chain (MHC) sarcomere protein expression was found in newly formed myotubes but not in cultures co-treated with PI3-K inhibitor (S5 and S6 Figs). Actually, insulin encouraged DNA synthesis (mitogenicity), and as long as myoblasts divide, MyoD protein content is detected. C2C12 muscle cells do not fully differentiate into myotubes. A small fraction remains mononuclear and doesn’t fuse. This tiny portion of cells might explain why MyoD expression was still present even though AKT- or MEK-mediated signaling was abrogated. In the intact C2C12 muscle cells, the most likely signaling pathways stimulated by insulin (depending on cell differentiation status) would be PI3-K/PKB/AKT or Ras/Raf/MEK/MAPK ERK1/2 or both [9, 47–49]. To stop the activity of a particular signaling pathway specific metabolic inhibitors were used, LY294002 for PI3-K/PKB/AKT or PD98059 to stop Ras/Raf/MEK/MAPK ERK1/2. Downstream steps (e.g. GSK-3β) could be blocked by LiCl or SB216763. AKT activity (phosphorylated at Thr^308^ by PDK1 and at Ser^473^ by mammalian target of rapamycin complex 2 –mTOR/rictor/LST8/GβL) is fundamental as this serine-threonine kinase activates mTOR complex 2 (mTOR/raptor/LST8/GβL) to stimulate eukaryotic initiation factor 2B (eIF2B) and ribosomal S6 kinase (S6K1) with resultant protein accretion. Whether GSK-3β in its active form (unphosphorylated at Ser^9^ and/or phosphorylated at Tyr^216^) has something to do with extensive protein degradation is a matter of debate, although AKT signaling and GSK-3β inhibitors prevent skeletal muscle protein degradation [50–52]. Activated Akt was known as capable not only of increasing the size of fibers in normal skeletal muscles but also of preserving muscle fiber size in muscles undergoing atrophy [53]. Akt seems to promote protein synthesis by phosphorylating GSK-3β, leading to its inhibition [54–55]. In contrast to insulin given alone where myogenin expression was distinctly elevated it almost utterly disappeared from blots after additional PI3-K inhibitor, but not upon PD98059 co-treatment with insulin (Fig 3A). Recently, ERK-mediated phosphorylation of Tfam was reported to downregulate mitochondrial transcription in SH-SY5Y cell line [56]. Furthermore, in several studies MEK inhibitor PD98059 was reported to stimulate myogenesis from C2C12 muscle cells [47–49, 57] pointing to switch from cell proliferation to cell differentiation that is associated with the withdrawal from cell cycle.

FOXO1 is transcription factor critical for skeletal muscle protein decay, as it controls the activity of atrogenes [58–59]. It is also among numerous molecular targets of insulin to withdraw FOXO1 from nuclear location and transcriptional activity [60]. Insulin reduced FOXO1 activity almost two-fold (Fig 2C, *p*<0.05). Blockade of PI3-K-mediated insulin signaling instantly restored FOXO1 transcriptional activity to control level (Fig 2D, *p*>0.05). Concomitant use of insulin and PD98059 initially inhibited FOXO1 activity, but this effect had vanished after 60 minutes of treatment (Fig 2E, *p*>0.05). The responses to metabolic inhibitors in the presence of IGF-1 advocate PI3-K as more important and potent FOXO1 inhibitor than MEK. In fact, these observations are consistent with previous reports pointing to PI3-K rather than MEK as the decisive kinase for inhibition of FOXO1 [26]. Moreover, at least in the liver FOXO1 integrates the insulin-dependent function of mitochondria [15]. The question then arises, whether this transcription factor is also involved in mitochondriogenesis in skeletal muscle cells?

We decided to decipher its role through quantitative estimation of genes known to control biogenesis of mitochondria and mitochondrial fusion *Mfn2*, *Cox-1*, *MtSSB* and *mtTFA* on the 1^st^, 3^rd^, and 5^th^ day of myogenesis. No doubt, insulin appeared a strong stimulus for these genes (Figs 4 and 5, *p*<0.01) however, to our surprise neither of pharmacological inhibitors administered separately (PI3-K, MEK, nor GSK-3β) could impede insulin-mediated effects (Figs 4 and 5, *p*>0.05). It is obvious, that some redundancy of insulin signaling circuits is in charge to regulate activity of selected mitochondria regulating genes in C2C12 myotubes. Calcineurin [61] and calmodulin-dependent protein kinase CaMK [62] are downstream of calcium signaling, MEF2 transcription factor nuclear and receptor coactivator PGC-1α [63]. In our experiment insulin concentration amounted to 10 nM (it is approximately from 12.5 to 100-times higher than normal insulin blood concentrations) so it can similarly to IGF-1 activate IGF-1 receptor and signaling pathway induced by calcium ion fluxes through the voltage-gated calcium channels [64]. We speculate that insulin-dependent mechanism of *Mfn2*, *Cox-1*, *MtSSB* and *mtTFA* activation on the 1^st^, 3^rd^, and 5^th^ day of myogenesis might be linked to calcineurin. In spite of a number of papers which address the importance of calcineurin in the control of skeletal muscle atrophy in cachexia great deal of results is limited in number and often inconsistent in merit and conclusions [65]. Anyway, there is compelling evidence that calcineurin variant inhibits FOXO activity and enhances skeletal muscle regeneration [66]. The emphasis is often put on neural control of muscle fibers during exercise, but one has to bear in mind that bioenergetics in growing skeletal muscle is tightly linked to mitochondria and oxidative metabolism. There is even general consensus that mitochondria control muscle growth and that this organelle is fundamental for skeletal muscle to sense insulin [67].

There is substantial evidence that mitofusin-2 (Mfn2) a mitochondrial fusion protein, stimulates respiration, substrate oxidation and OXPHOS subunits expression [57]. In turn, insulin resistance due to hyperglycemia or exposure to saturated fatty acids severely impairs mitochondrial functions [68]. Insulin-dependent rise in *mtTFA* expression was inhibited by GSK-3β blockers (LiCl and SB216763) also pointing to other than PI3-K/AKT signaling pathway. Combined PD98059 and insulin treatment amplified insulin-stimulating effect on mitochondriogenesis (increased *Mfn2*, *Cox-1* and *mtTFA* activity) which is in concert with our former observations regarding myogenesis in C2C12 muscle cells [9]. From this study we conclude, that inhibition of MAPK MEK accelerates differentiation of muscle cells together with biogenesis of mitochondria and that it is incompletely controlled by PI3-K/AKT. Essentially, mitochondria must display a retrograde signaling to regulate their own biogenesis [36, 69]. Accordingly, insulin-mediated GSK-3β phosphorylation at serine 9 remained intact after PD98059 (MEK inhibitor) addition, whereas it instantly dropped after LY294002 (PI3-K inhibitor) treatment (Fig 6). ERK1/2, the substrate of MEK undergoes phosphorylation at T202/Y204, and as we reported previously [70], MEK enables GSK-3β activation through phosphorylation of its domain at tyrosine 216. Consequently, GSK-3β phosphorylation status might be crucial for the switch from the proliferation (active) into differentiation phase (inactive).

As mentioned, insulin stimulated several genes selected for their significance in mitochondriogenesis and oxidative metabolism (Figs 4 and 5, *p*<0.01). This scrutiny, however, was not entirely matched by the protein content of the respective translation products. Certainly, insulin stimulated Mfn-2 and COX IV protein expression and this rise was further elevated by concomitant use of LiCl but not SB216763 GSK-3β inhibitor (Fig 7). Although it is not clear whether the inhibition of FOXO1 by insulin or LiCl directly leads to the increase of COX-1 and COX IV proteins the fusion indices and myosin sarcomere expression levels were markedly elevated every time insulin or LiCl or both were administered (S5 and S6 Figs). These observations indicate that insulin not only controls glucose homeostasis by targeting FOXO1 and vice versa (insulin receptor modulation) but this evolutionary conserved mechanism is also indispensable for skeletal muscle with important input from mitochondria. Again GSK-3β is indicated as important player to manage translation of two presumably decisive proteins for mitochondria. Notably, the insulin effect was capably blocked by simultaneous presence of LY294002, positioning PI3-K/AKT at the upstream level to control protein synthesis of Mfn-2 and COX IV (Fig 7). Similar testimony was reported by other investigators [11, 57, 67–68, 71]. Pivotal role of PI3-K/AKT in the translational control was further substantiated by an additional rise in Mfn-2 and COX IV expressions after concomitant use of LiCl but not SB216763 (Fig 7). In this point, it should be stressed that amplification of the insulin-mediated increase in protein content of Mfn-2 and COX IV by LiCl reminds us that S9 phosphorylation of GSK-3β and subsequent drop in activity of this kinase is a subject of “fine tuned” modulation. Therefore, insulin does not completely block GSK-3β activity, as LiCl can further increase protein expression of Mfn-2 and COX IV (Fig 7). To our knowledge, this is the first report which points to the possible role of GSK-3β in the integration of insulin effect with mitochondrial function. A great body of evidence is related to the possible contribution of GSK-3β to skeletal atrophy/cachexia atrophy without focusing on the integration with mitochondria [46, 72]. In this experiment we showed that GSK-3β inhibits translation of some proteins critical for mitochondrial function but this kinase is less efficient in transcriptional control of genes imperative for coordinated expression of gene templates for nuclear and mitochondrial proteins.

To clear the issue whether GSK-3β integrates insulin and mitochondrial signaling we quantitatively examined the interaction of this kinase with Mfn-2 protein at the 3^rd^ day of myogenesis, the most critical for fusion of C2C12 myoblasts. In untreated cells (control conditions) both proteins were bound together forming a complex that was affected neither by insulin, nor PI3-K inhibitor LY294002 (Fig 8). Nonetheless, fewer Mfn-2 was bound to GSK-3β when MEK inhibitor (PD98059) was added to the medium. It suggests that inhibition of MEK (possible activator of GSK-3β) might lead to drop of GSK-3β activity and GSK-3β is engaged in the assembly of these protein partners. Actually, co-treatment of C2C12 muscle cells with insulin and PD98059 decreased expression of P-T202/Y204 ERK1/2 (specific target of MEK) at the same, and during following days (3–5). Given that MEK activity was reduced upon PD98059 co-treatment as corroborated by western blotting (Fig 7), presumably active GSK-3β represses Mfn-2 from its action on mitochondriogenesis, but once inactivated (drop in MEK activity) it can contribute (initiate?) muscle differentiation program.

Summing up, inhibitors of PI3-K/AKT/GSK-3β but not that of Ras/Raf/MEK/ERK1/2 abolished insulin-induced viability, mitogenesis and ATP synthesis during skeletal muscle cell differentiation in normoglycemic conditions. Insulin-dependent myogenesis was accompanied by the rise in *mtTFA*, *MtSSB*, *Mfn2*, and mitochondrially encoded *Cox-1* gene expressions and elevated level of proteins which control functions of mitochondria (PKB/AKT, Mfn-2). Insulin, in the PI3-K/AKT-dependent manner reduced FOXO1 activity and altered GSK-3β phosphorylation status. Additionally, the inhibition of GSK-3β activity by the specific metabolic inhibitor LiCl (but not SB216763) raised expression of genes and proteins central for metabolic activity of mitochondria. In turn, lack of GSK-β phosphorylation (P-S9) on serine 9 (essential for inactivation) led to the reduction in Mfn2 and COX IV protein expressions. Some of insulin related effects (myogenesis) were not entirely dependent on PI3-K/AKT/GSK-3β or Ras/Raf/MEK/ERK1/2 activation. Also inhibitors of mitochondrial respiration (rotenone) and ATP synthase (oligomycin) were shown to impair insulin-dependent effects. Overall, the results of this study indicate, that there are at least two main targets in insulin-mediated myogenesis: notably transcriptional factor FOXO1 and GSK-3β. In the elegant study conducted by Puig and Tjian [25] mammalian cells up-regulated the insulin receptor mRNA in the absence of serum, conditions that induce the dephosphorylation and activation of FOXO1. Interestingly, insulin is able to reverse this effect. Our observations indicate that upon insulin administration both increased P-S9 GSK-3β and inhibited FOXO1 activity augmented viability, mitogenicity, and ATP synthesis, resulting in elevated myotube formation.

## Supporting Information

S1 Fig The influence of metabolic inhibitors on long-term insulin-dependent cell viability. Long term effects of insulin (10 nM) alone or given together with selected metabolic inhibitors (at indicated concentrations) on cell viability measured with MTT (a, b, c, d, e) and Neutral Red assays (f, g, h, i, j) during 5 subsequent days of myogenic differentiation. Regression analysis (4^th^ order polynomial). The results are indicative of three independent experiments performed in eight replicates.
(TIF)

S2 Fig The influence of metabolic inhibitors on long-term insulin-dependent myotube formation. Myotube formation from C2C12 myoblasts. Muscle cell phenotype was monitored in phase-contrast microscope (magnification 1x5000). Monolayers were photographed at day 1, 3, 5, of differentiation process. Horizontal panels of photographs from top to bottom: CTRL [untreated cells], insulin [10 nM], insulin + PD98059, insulin + LY294002 (at indicated concentrations) at following days.
(TIF)

S3 Fig The influence of metabolic inhibitors on long-term insulin-dependent myotube formation. Myotube formation from C2C12 myoblasts. Muscle cell phenotype was monitored in phase-contrast microscope (magnification 1x5000). Monolayers were photographed at day 1, 3, 5, of differentiation process. Horizontal panels of photographs from top to bottom: CTRL [untreated cells], insulin [10 nM], LiCl, insulin + LiCl (at indicated concentrations) at following days.
(TIF)

S4 Fig The influence of metabolic inhibitors on long-term insulin-dependent myotube formation verified with fusion index. Bar chart (mean ± SEM) representing fusion index: the relation between the number of nuclei (>3) within myotubes and the average of the whole number of nuclei multiplied by 100% at day 1, 3, and 5 of myotube formation from C2C12 myoblasts. The results are indicative of three independent experiments. Statistical differences from non-treated cultures (2% HS/DMEM) within each day are indicated by asterisks (*p<0.05; **p<0.01; ***p<0.001), whereas statistical differences between the treatments and untreated control cells within each day are ticked with different lower case letters.
(TIF)

S5 Fig Cytoimmunofluorescence. **The influence of metabolic inhibitors on long-term insulin-dependent myosin sarcomere expression.** Myotube formation from C2C12 myoblasts. Myosin sarcomere expression envisaged by cytoimmunofluorescence. Myosin is visible in green and nuclei in blue (magnification 1x20000). Monolayers were photographed at day 1, 3, 5, of differentiation process. Horizontal panels of photographs from top to bottom: CTRL [untreated cells], insulin [10 nM], insulin + PD98059, insulin + LY294002 (at indicated concentrations) at following days.
(TIF)

S6 Fig Cytoimmunofluorescence. **The influence of metabolic inhibitors on long-term insulin-dependent myosin sarcomere expression.** Myotube formation from C2C12 myoblasts. Myosin sarcomere expression envisaged by cytoimmunofluorescence. Myosin is visible in green and nuclei in blue (magnification 1x20000). Monolayers were photographed at day 1, 3, 5, of differentiation process. Horizontal panels of photographs from top to bottom: CTRL [untreated cells], insulin [10 nM], LiCl, insulin + LiCl (at indicated concentrations) at following days.
(TIF)
