# Supplementary figures and images for: FOXO1 and GSK-3β Are Main Targets of Insulin-Mediated Myogenesis in C2C12 Muscle Cells
Source: PLoS One. 2016 Jan 19;11(1):e0146726. doi: 10.1371/journal.pone.0146726 (PMC4718532; doi:10.1371/journal.pone.0146726)

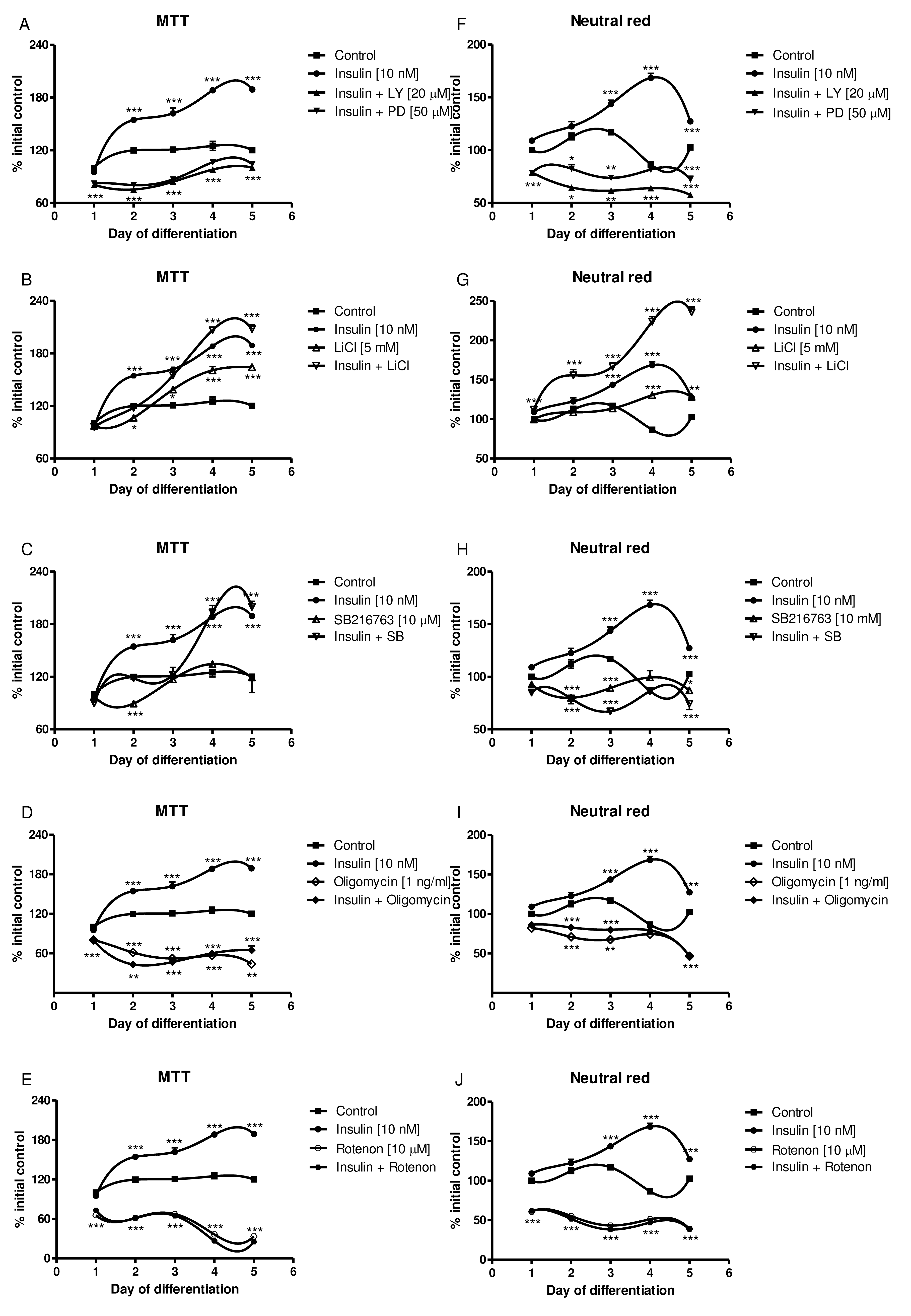

Supplement: S1 Fig — Long term effects of insulin (10 nM) alone or given together with selected metabolic inhibitors (at indicated concentrations) on cell viability measured with MTT (a, b, c, d, e) and Neutral Red assays (f, g, h, i, j) during 5 subsequent days of myogenic differentiation. Regression analysis (4th order polynomial). The results are indicative of three independent experiments performed in eight replicates. (TIF) [file pone.0146726.s001.tif]

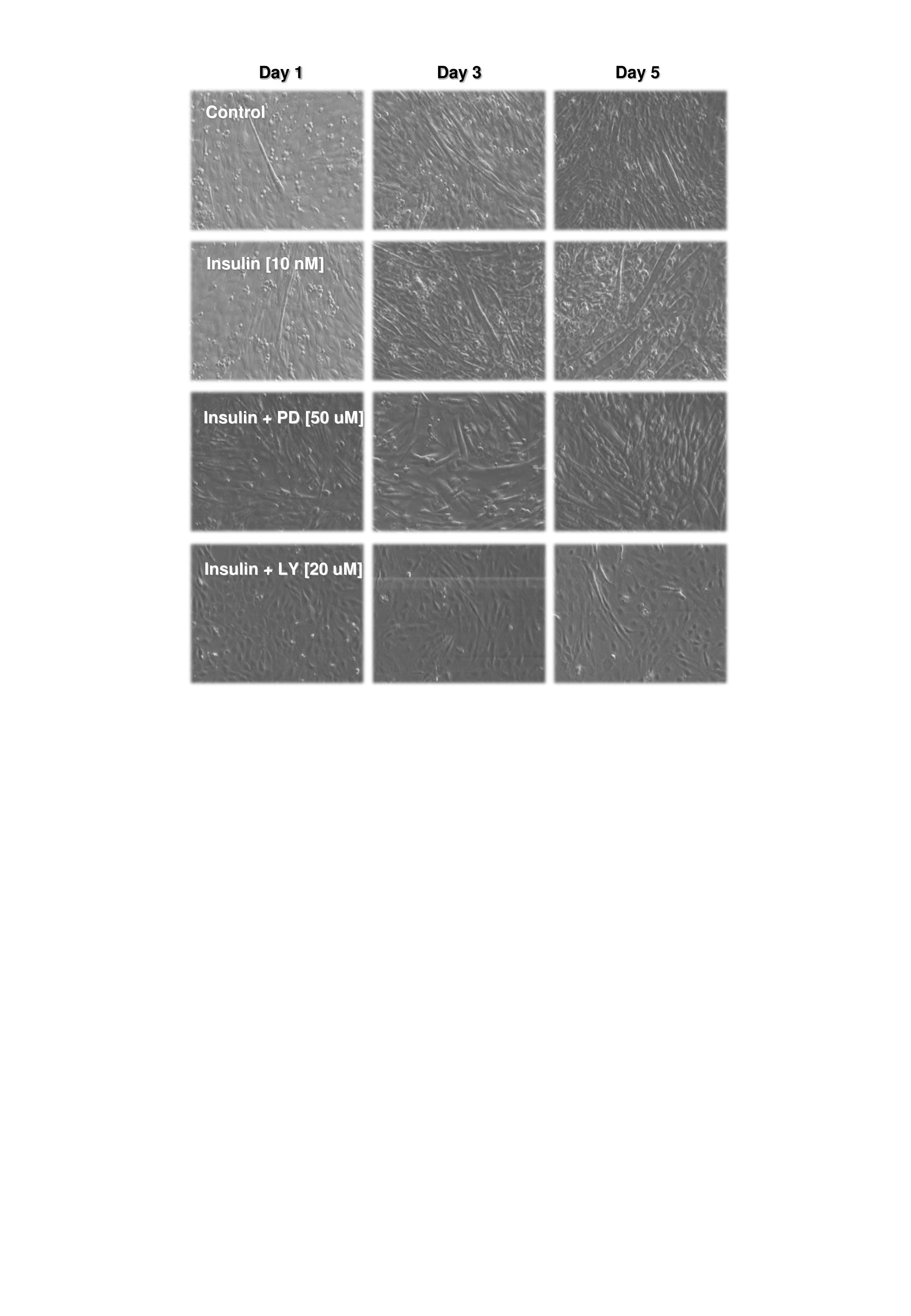

Supplement: S2 Fig — Myotube formation from C2C12 myoblasts. Muscle cell phenotype was monitored in phase-contrast microscope (magnification 1x5000). Monolayers were photographed at day 1, 3, 5, of differentiation process. Horizontal panels of photographs from top to bottom: CTRL [untreated cells], insulin [10 nM], insulin + PD98059, insulin + LY294002 (at indicated concentrations) at following days. (TIF) [file pone.0146726.s002.tif]

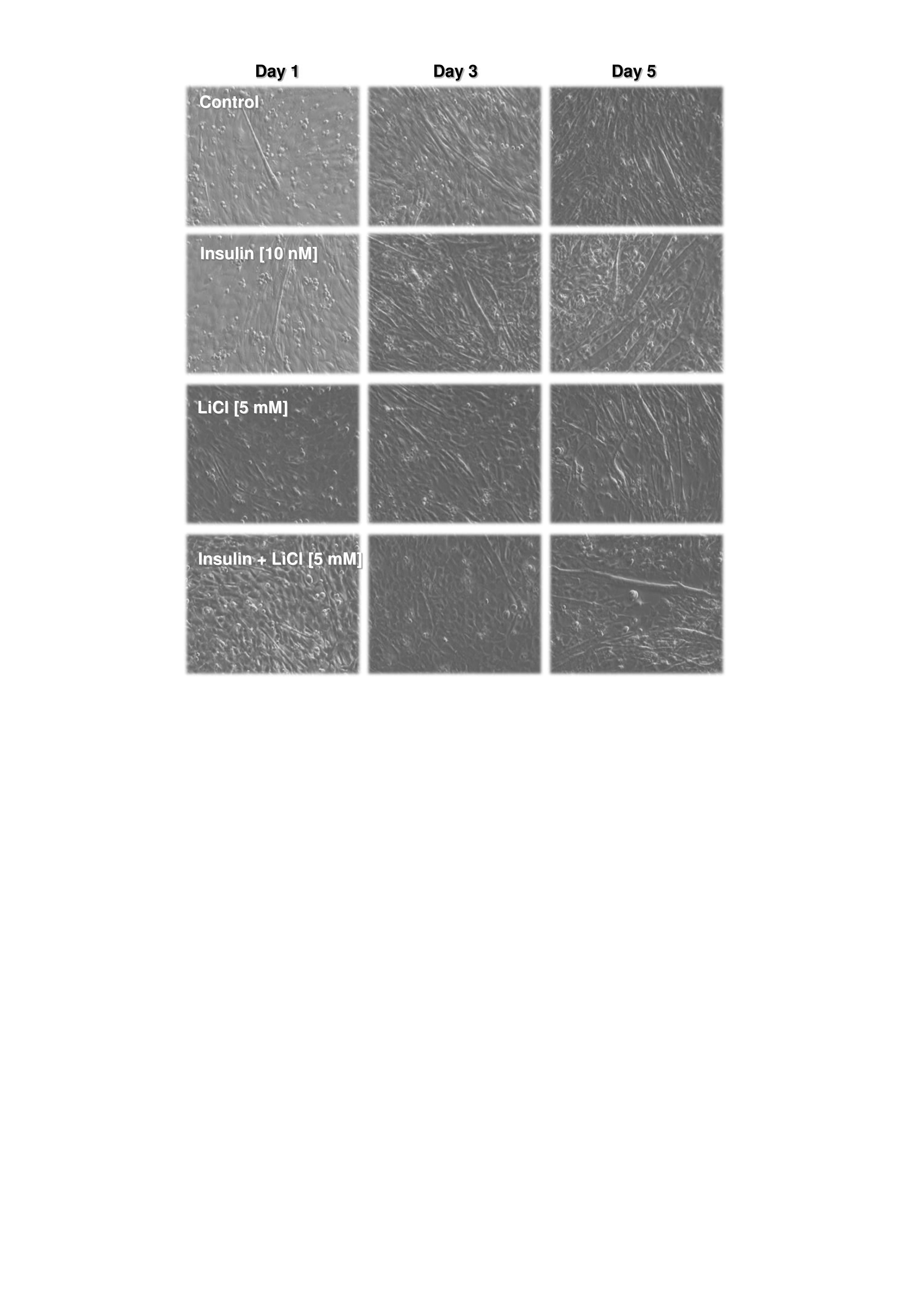

Supplement: S3 Fig — Myotube formation from C2C12 myoblasts. Muscle cell phenotype was monitored in phase-contrast microscope (magnification 1x5000). Monolayers were photographed at day 1, 3, 5, of differentiation process. Horizontal panels of photographs from top to bottom: CTRL [untreated cells], insulin [10 nM], LiCl, insulin + LiCl (at indicated concentrations) at following days. (TIF) [file pone.0146726.s003.tif]

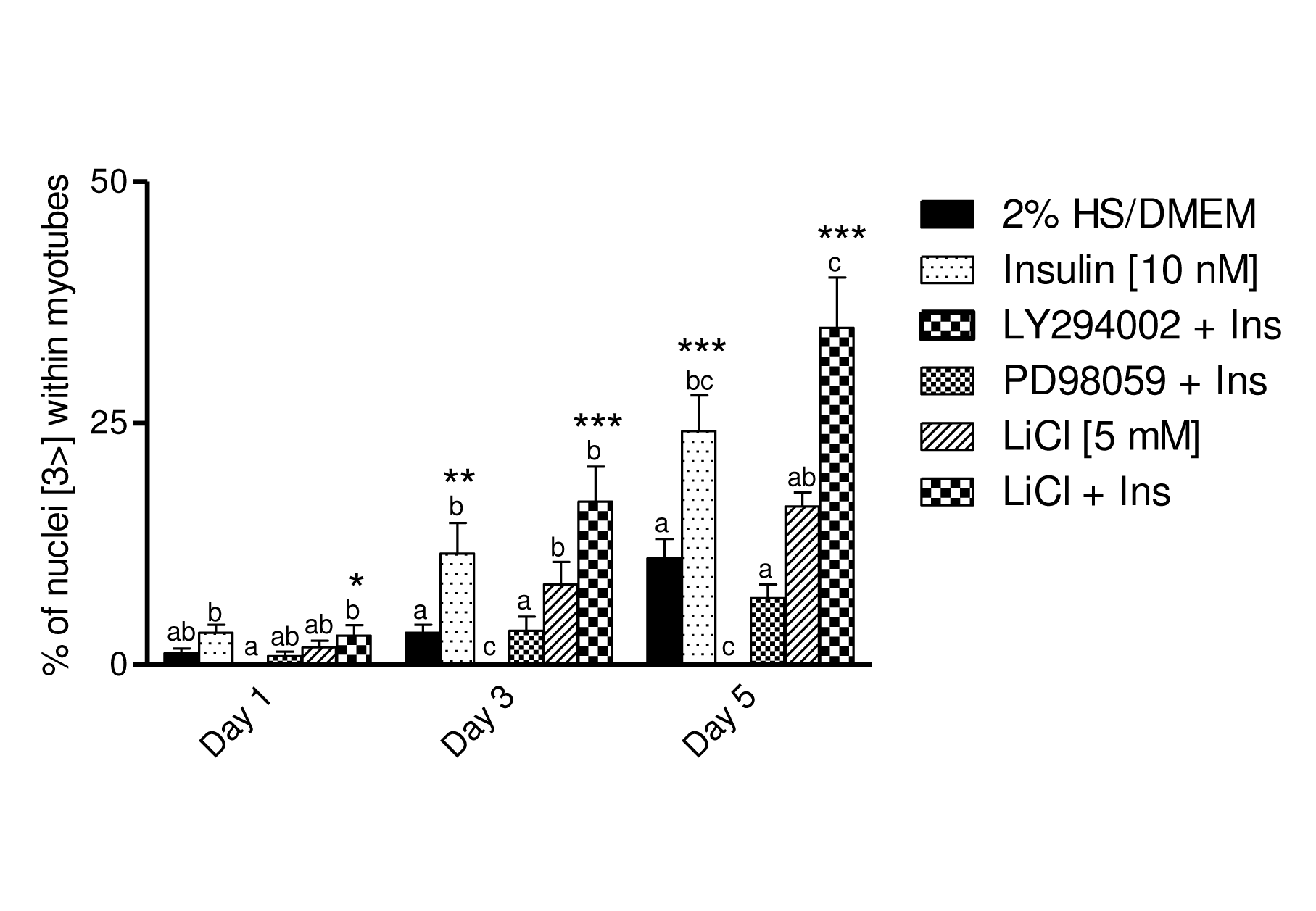

Supplement: S4 Fig — Bar chart (mean ± SEM) representing fusion index: the relation between the number of nuclei (>3) within myotubes and the average of the whole number of nuclei multiplied by 100% at day 1, 3, and 5 of myotube formation from C2C12 myoblasts. The results are indicative of three independent experiments. Statistical differences from non-treated cultures (2% HS/DMEM) within each day are indicated by asterisks (*p<0.05; **p<0.01; ***p<0.001), whereas statistical differences between the treatments and untreated control cells within each day are ticked with different lower case letters. (TIF) [file pone.0146726.s004.tif]

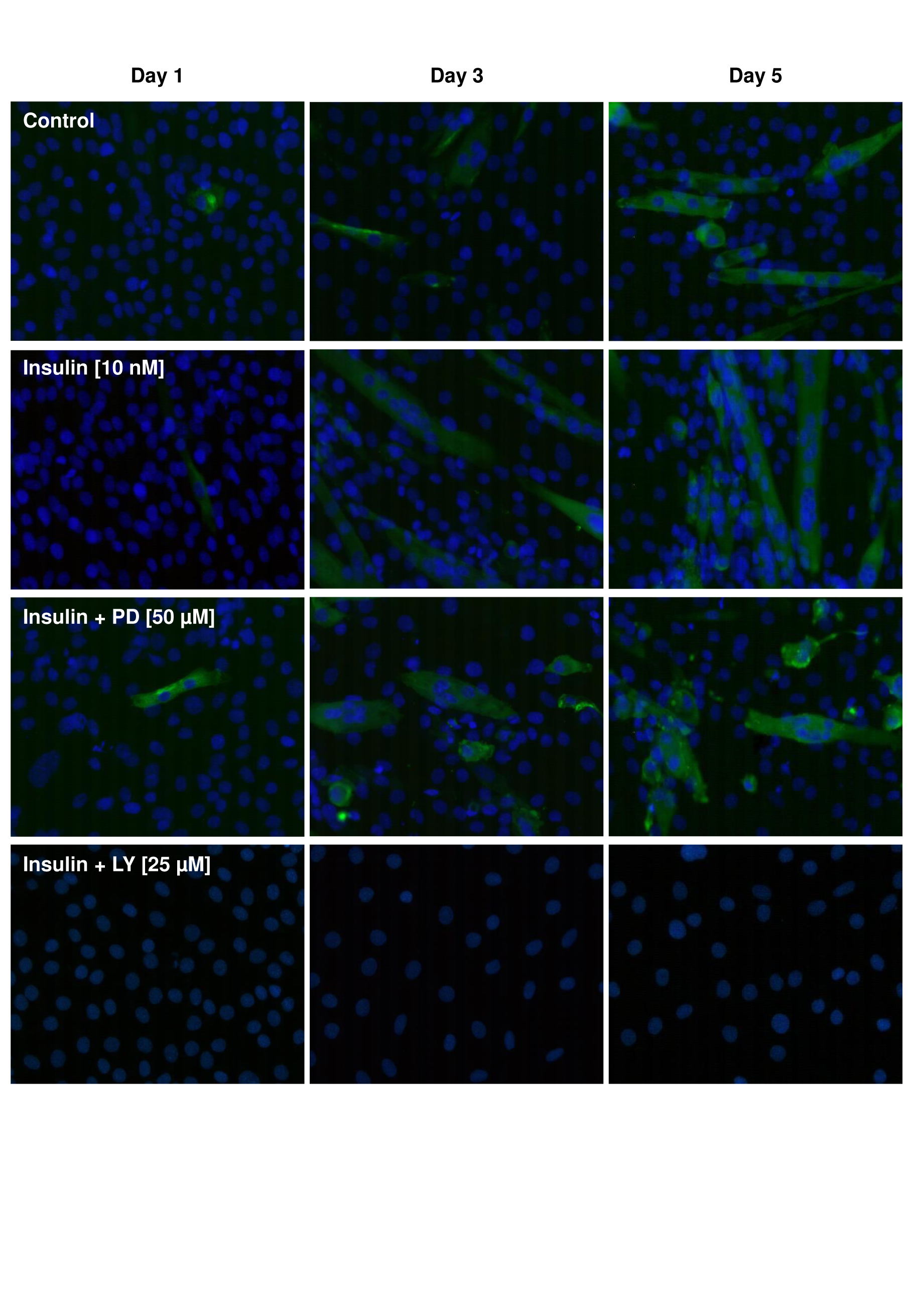

Supplement: S5 Fig — The influence of metabolic inhibitors on long-term insulin-dependent myosin sarcomere expression. Myotube formation from C2C12 myoblasts. Myosin sarcomere expression envisaged by cytoimmunofluorescence. Myosin is visible in green and nuclei in blue (magnification 1x20000). Monolayers were photographed at day 1, 3, 5, of differentiation process. Horizontal panels of photographs from top to bottom: CTRL [untreated cells], insulin [10 nM], insulin + PD98059, insulin + LY294002 (at indicated concentrations) at following days. (TIF) [file pone.0146726.s005.tif]

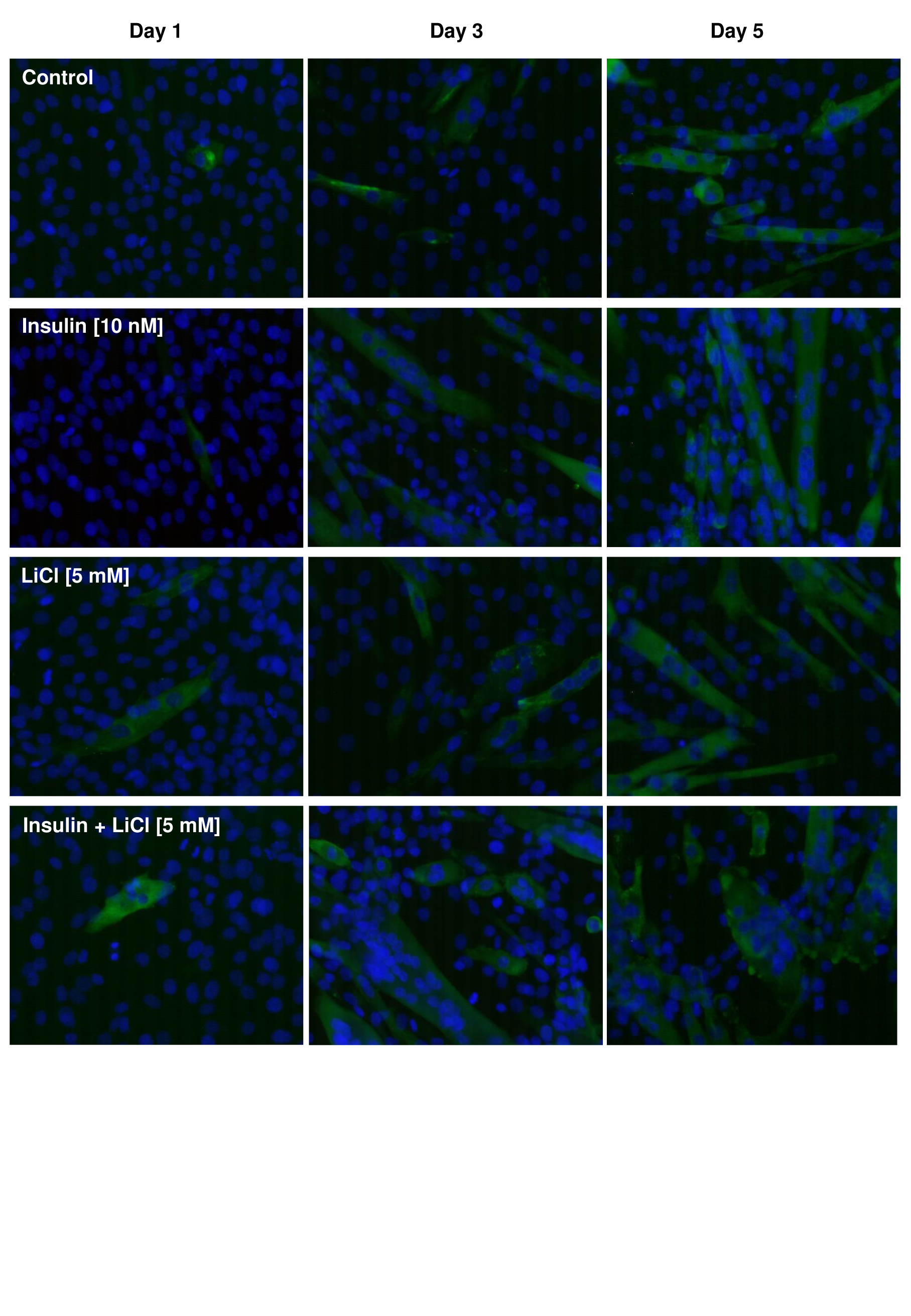

Supplement: S6 Fig — The influence of metabolic inhibitors on long-term insulin-dependent myosin sarcomere expression. Myotube formation from C2C12 myoblasts. Myosin sarcomere expression envisaged by cytoimmunofluorescence. Myosin is visible in green and nuclei in blue (magnification 1x20000). Monolayers were photographed at day 1, 3, 5, of differentiation process. Horizontal panels of photographs from top to bottom: CTRL [untreated cells], insulin [10 nM], LiCl, insulin + LiCl (at indicated concentrations) at following days. (TIF) [file pone.0146726.s006.tif]
